# *Eleutherococcus senticosus* (*Acanthopanax senticosus*): An Important Adaptogenic Plant

**DOI:** 10.3390/molecules30122512

**Published:** 2025-06-08

**Authors:** Grzegorz Kos, Katarzyna Czarnek, Ilona Sadok, Agnieszka Krzyszczak-Turczyn, Paweł Kubica, Karolina Fila, Gizem Emre, Małgorzata Tatarczak-Michalewska, Małgorzata Latalska, Eliza Blicharska, Daniel Załuski, Nazım Şekeroğlu, Agnieszka Szopa

**Affiliations:** 1 Department of Medicinal Plant and Mushroom Biotechnology, Faculty of Pharmacy, Jagiellonian University, 9 Medyczna St., 30-688 Kraków, Poland; grzegorz.kos@student.uj.edu.pl (G.K.); p.kubica@uj.edu.pl (P.K.); 2 Institute of Medical Science, Faculty of Medical, The John Paul II Catholic University of Lublin, Konstantynów 1 H St., 20-708 Lublin, Poland; 3 Department of Biomedical and Analytical Chemistry, Institute of Biological Sciences, Faculty of Medicine, Collegium Medicum, The John Paul II Catholic University of Lublin, Konstantynów 1J St., 20-708 Lublin, Poland; ilona.sadok@kul.pl (I.S.); agnieszka.krzyszczak@kul.pl (A.K.-T.); 4 Institute of Agrophysics, Polish Academy of Sciences, Doświadczalna 4 St., 20-290 Lublin, Poland; k.fila@ipan.lublin.pl; 5 Department of Pharmaceutical Botany, Faculty of Pharmacy, University of Marmara, İstanbul 34854, Türkiye; gizem.bulut@marmara.edu.tr; 6 Department of Pathobiochemistry and Interdisciplinary Applications of Ion Chromatography, Medical University of Lublin, 1 Chodźki St., 20-093 Lublin, Poland; malgorzata.tatarczak-michalewska@umlub.pl (M.T.-M.); eliza.blicharska@umlub.pl (E.B.); 7 Chair and Department of General and Pediatric Ophthalmology, Medical University of Lublin, 20-079 Lublin, Poland; malgorzata.latalska@umlub.pl; 8 Department of Pharmaceutical Botany and Pharmacognosy, Ludwik Rydygier Collegium Medicum, Nicolaus Copernicus University, 9 Marie Curie-Skłodowska St., 85-094 Bydgoszcz, Poland; daniel.zaluski@cm.umk.pl; 9 Department of Biology, Faculty of Science and Literature, Gaziantep University, Gaziantep 27310, Türkiye; nazimsekeroglu@gantep.edu.tr

**Keywords:** Siberian ginseng, utility of *Eleutherococcus senticosus*, adaptogenic raw materials, phytochemical composition, biological activity, medicinal plants

## Abstract

This comprehensive review focuses on *Eleutherococcus senticosus* (ES), examining the phytochemical composition, traditional medicinal roles, ecological traits, and pharmacological effects. Native to Northeast Asia, ES is used in traditional Chinese, Korean, and Japanese medicine. The rhizomes and bark are utilized medicinally and valued for their adaptogenic properties that enhance stress resistance, boost mental and physical endurance, and exhibit immunostimulatory effects that strengthen the immune system. Its pharmacological potential stems from a variety of bioactive compounds, including eleutherosides, lignans, saponins, flavonoids, and polysaccharides, which contribute to health benefits such as neuroprotective, antidiabetic, anticancer, and antioxidative activities. Neuroprotective properties may aid in the management of neurodegenerative conditions, such as Alzheimer’s and Parkinson’s disease, while antidiabetic effects support glucose regulation and insulin sensitivity. With increasing demands and conservation concerns, sustainable cultivation practices are essential, as ES is endangered in some areas. Plant biotechnology techniques offer solutions to enhance secondary metabolite yields while ensuring genetic stability and minimizing environmental impacts. ES is a promising natural resource for various industries because of its extensive benefits. Still, its conservation and sustainable production are critical and require ongoing research and innovative cultivation strategies.

## 1. Introduction

*Eleutherococcus senticosus* (Rupr. et Maxim.) Maxim., thereafter ES, is also under synonymous name - *Acanthopanax senticosus* (Rupr. et Maxim.) Harms and belongs to the family Araliaceae. In Europe and North America, it is known by the common name “Siberian ginseng”. Traditionally, ES is called “ginseng” despite not being a member of the *Panax genus*. This is because of the similar appearance and use of raw materials to other plants of the *Panax genus* [1]. The term “Siberian” is probably related to the fact that initially the raw material for research was obtained mainly from Siberia [2]. In traditional Chinese medicine (TCM), it is called “Ciwujia”, while in Korean medicine, it is known as “Gasiogapi” [3,4,5]. Extracts from the raw material ES exhibit a wide range of pharmacological activities. Intensive research on this plant has allowed for the expansion of knowledge regarding its health-promoting properties, which go beyond its traditional uses.

According to the guidelines of the European [6], Korean [7], and Japanese Pharmacopoeias [8], the raw material of ES is the rhizome (*Eleutherococcisenticosirhizoma*), permitted for use with roots or the bark of the underground parts alone (*Acanthopanacis cortex*).Similar raw materials are indicated in the monographs of the World Health Organization (WHO) [9] and the European Medicines Agency (EMA) [10]. The raw material is standardized for eleutheroside content. According to the requirements of the European Pharmacopoeia, the total content of eleutheroside B and eleutheroside E should be at least 0.08% of the dry weight (DW) of the raw material [6]. In addition to the underground parts of ES, fruit extracts are increasingly used. Moreover, herbal teas are produced from dried leaves [11,12]. Moreover, ES is authorized for use as a calming and skin-improving ingredient in cosmetic products. Such properties are attributed to it by the European Cosmetic Ingredients Database (Cosing).

## 2. Ecological and Botanical Characteristics

The family to which ES belongs, the Araliaceae, includes 84 genera that are native to Asia, the Malay Peninsula, Polynesia, Europe, North Africa, and America [13]. The natural habitat of ES is located in Northeast Asia. ES occurs mainly in the Korean Peninsula, Japan, northeastern China, and the eastern coast of Russia [14].

This species inhabits the undergrowth of mountain-mixed and coniferous forests [4]. “*Eleutherococcus*” (from the Greek eleutheros meaning ‘free’ and kokkos meaning ‘pip’ or ‘seed’ more precisely a pyrene in botanical terminology) is a thorny shrub. The thorniness, reflected by the specific epithet “*senticosus*” an adjective meaning in Latin ‘full of briers or thorns’, has led to the common names in Russian of ‘thorny *Eleutherococcus*’ (eleutherokokkkoljuchii), ‘untouchable’ (nedotroga), ‘devil’s bush’ (dyavol’skiikust), ‘wild pepper’ (dikiiperets), or even ‘thorny bearer of free berries’ (svobodnojagodnikkoljuchii) [15].

ES is a shrubby plant that grows to about 2.5 m in height. It has a woody stem that is covered with thin thorns and grows from leaf petioles. Palmately compound leaves have slender petioles, 3–12 cm long, with sparse pricklets, and 3–5 leaflets. The leaflets are elliptical, obovate, or oblong, 5–13 cm long, and 3–7 cm wide [16].

Its white flowers are arranged in an umbel-type inflorescence. The fruits are black drupes. The above-ground shoots grow from a strongly branched rhizome 15–30 cm long and 1–2.5 cm in diameter. Under natural conditions, the plant blooms from June to July and bears fruits from August to October [3,8,17].

In Northeast China, ES is classified as one of the main woody plants producing non-timber forest products (NTFPs), which are used for household livelihoods, economic income, and culture. This economically important forest tree is a medicinal food homology plant that is widely popular, and its population is dramatically decreasing. Additionally, it is caused by climate change such as climate warming and intensified human interventions. The natural habitats of ES have shifted toward higher latitudes and elevations. According to the latest results, the suitable habitat area of ES in China is 123,449 km^2^, which will decrease in the 2090s.

Currently, raw materials for ES are mainly obtained from natural sites. Owing to the increasing demand for products containing ES, its natural resources are being depleted. In Korea, this species has already been listed in the Red List of Endangered Species [18]. Therefore, attempts are being made to cultivate ES, mainly in research centers, especially in China and South Korea, and even in Europe (Poland) [17,19]. The main obstacle in the traditional cultivation of ES is the slow process of seed germination. Large-scale artificial cultivation would allow for the protection of the natural sites of this plant. ES can be propagated in two ways: through seeds or stem cuttings. Unfortunately, ES has low reproductive capacity, and both generative and vegetative reproduction are ineffective [20,21,22]. Seed germination under natural conditions is poor, depends on many factors, and is possible after several years of stratification [20,22]. In vegetative propagation, only underground organs can be effectively used; in particular, root fragments with originally developed shoots (induction of rooting of shoot fragments is very difficult), but obtaining cuttings in this manner on a large scale is not very effective [20,21]. Therefore, there is great hope associated with plant biotechnology techniques and in vitro cultures of ES [23].

## 3. Traditional Applications

ES is known in TCM as well as in traditional Korean and Japanese medicine, and its medical use in Asia has persisted for over 2000 years [24]. The rhizome (*Eleutherococcisenticosirhizoma*), fruit, and leaf are the main components of herbal medicines.ES is considered a *panacea*. Prophylactic use is recommended to prevent disease and maintain good general health. It is also a known adaptogen used to treat physical and mental exhaustion and exposure to long-term stress. This activity is described as “strengthening the Qi”, as Qi is the name for vital energy in TCM [25]. ES is also valued for its immunostimulatory and antiaging effects [25,26]. The immunostimulatory effect is described in TCM as “fortifying the spleen” [14]. Its traditional use is known for its effects on rheumatic pain and bone disease. According to the Chinese Pharmacopoeia, it can also be used to alleviate symptoms of cerebral ischemia associated with cerebral arteriosclerosis and cerebral thrombosis [27]. Moreover, it is often used as the main ingredient in herbal mixtures. Common compositions include different parts of ES combined with plants such as *Codonopsis pilosula*, *Schisandra chinensis*, and *Polygala sibirica* [14].

ES is also particularly valued in Russian ethnomedicine, where it is used as an immunostimulatory dietary supplement [12]. Its popularity dates back to the Union of Soviet Socialist Republics (USSR) period, when it was popularized by state authorities as an adaptogen [2].

## 4. Chemical Composition

### 4.1. Key Bioactive Constituents

The first documented studies on the chemical composition of ES were published in the late 1960s and 1970s [15]. However, even in recent decades, a large number of chemical studies on ES have been published. They have concentrated on the isolation and identification of various compounds specific to ES. Currently, new compounds characteristic of ES have been identified.

**Figure 1:**
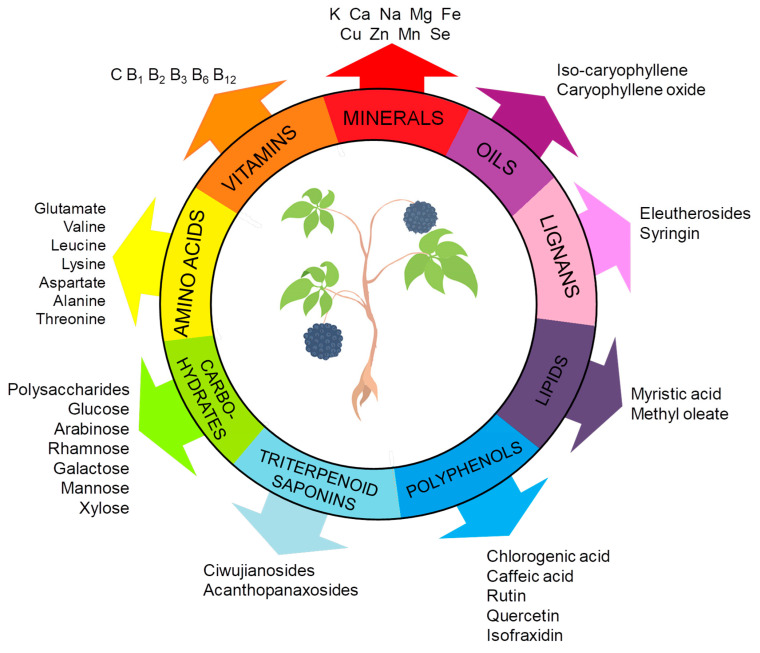
Key bioactive constituents of ES. The figure was prepared using Microsoft Office PowerPoint 2007.

**Figure 2:**
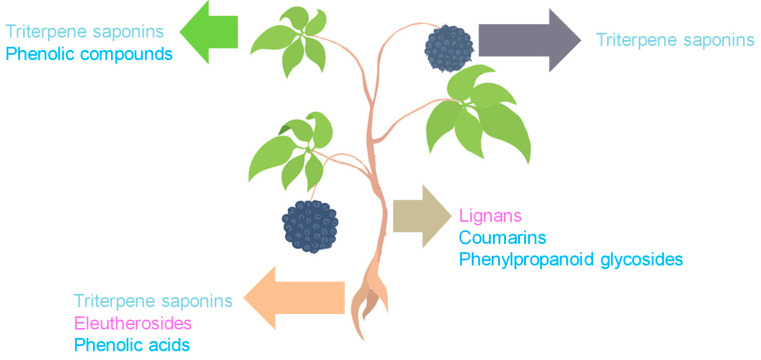
Distribution of major bioactive constituents of ES by plant parts (the pink color indicates compounds classified as lignans, the light blue color indicates triterpene saponins, and the dark blue color indicates phenolic compounds). The figure was prepared using Microsoft Office Power Point 2007.

ES constitutes a complex matrix, and the following groups of compounds can be distinguished: volatile compounds (essential oils), lignans, triterpenoid saponins, coumarins, flavones, carbohydrates and polysaccharides, vitamins, amino acids, lipids, and minerals (Figure 1). They occur unevenly in different parts of the plant (Figure 2). For example, triterpene saponins are the main components of leaves, fruits, shoots, and roots [28]. In contrast, stems are rich in lignans, coumarins, and phenylpropanoid glycosides [29]. Phenolic acids are more abundant in the leaves than in the stems [28]. Many of the ES components are biologically active, such as eleutherosides, isofraxidin, syringin, lignans, chiisanoside, acanthoside, senticoside, *β*-sitosterol, sesamine, and savinine [21,30,31].

### 4.2. Lignans and Eleutherosides

**Figure 3:**
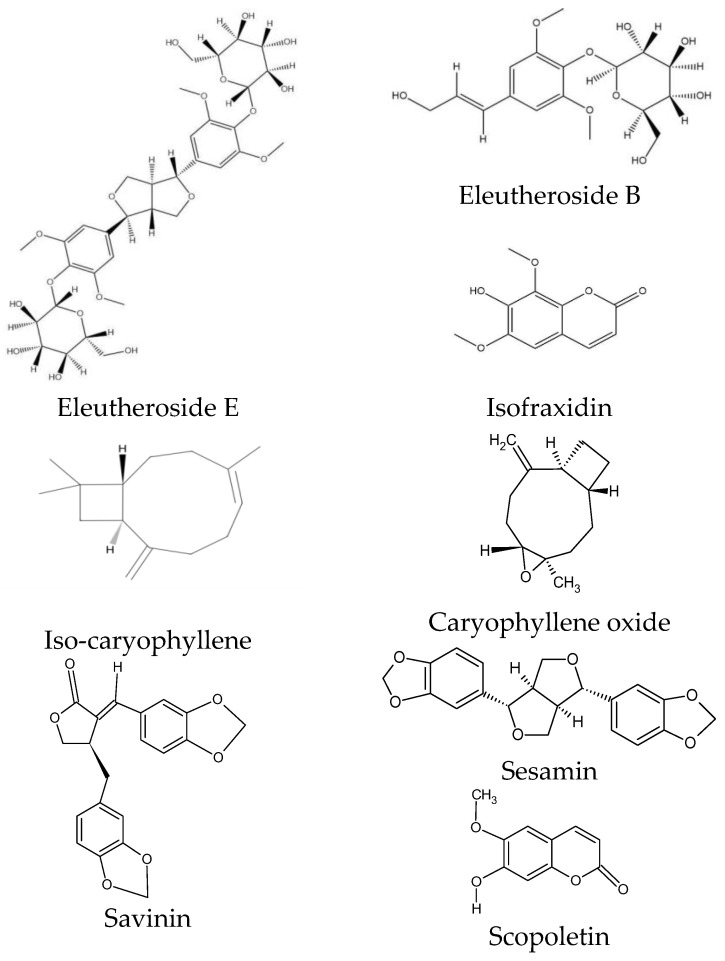
Chemical structure of phytochemicals characteristic of ES.

ES is characterized by a specific chemical composition. Eleutherosides are the most valuable group of compounds, occurring at the highest concentrations in the rhizome. This group of secondary metabolites belongs to glycosides and is considered the main component of ES with pharmacological effects [31]. Eleutherosides include compounds that differ significantly in their chemical structures. They consist of amino sugars and uronic acids bonded with non-sugar substances (aglycones or ligands) through terminal carbon atoms [31]. These phytochemicals are divided into two subgroups: glycoside derivatives of triterpene saponins (eleutherosides I, K, L, and M) and phenylpropane derivatives (eleutherosides B, D, and E) [26]. Among the above compounds, the most important in terms of biological activity are eleutherosides B and E [32], whose structures are presented in Figure 3.

Eleutheroside B is the same as syringin—a glycoside with sinapyl alcohol as its aglycone [33]. Eleutheroside E (syringaresinol), on the other hand, belongs to the lignan family and is a compound specific to ES [34,35].

The eleutheroside distribution varies throughout the plant. Their contents in the stems are typically higher than those in the roots and leaves [31]. In the fruits of ES, the total yield of eleutheroside B, eleutheroside E, and isofraxidin was estimated at around 30 mg/g [36]. Table 1 summarizes the available data on the contents of individual eleutherosides throughout the plant.

Despite the presence of eleutherosides, other minor lignans are present in ES. This plant synthesizes sesamin, isomaltol 3-O-α-D-glucopyranoside, and thymidine [37]. Moreover, the plant can produce and accumulate lignan precursors, such as hydroxycinnamic acid-caffeic acid and coniferyl aldehyde [27]. Many known lignans have been identified in ES; however, new compounds have also been isolated from the plant. For example, Wei et al. isolated a new lignan—1,6-bis-(3-methoxy-4-hydroxyphenyl)hexane-1,6-dione [38], and Feng et al. isolated a new neo-lignan named (7′S,8′R)-4′,5′,9′-trihydroxy-5-methoxy-4,8′-oxyneolign-7-en-9-al from the ethyl acetate-soluble extract of ES [39].

### 4.3. Coumarins

Among the coumarins, isofraxidin, isofraxidin-7-O-D-glucoside, and scopoletin were found in ES [27]. Isofraxidin (Figure 3) is also found in plants of the *Fraxinus* and *Artemisia* genera, but its concentration in the raw material of ES is one of the highest [40,41]. Coumarins also accumulate in plant stems [28].

**Table 1:** Contents of bioactive compounds isolated from different plant parts of ES.

Compound	Amount (mg/g DW)					References
	Whole Plant	Leaves	Roots	Fruits	Stems	
**Eleutherosides and lignans**						
Eleutheroside B	0.07–13.55	NA	NA	NA	NA	[42]
	<LOD–0.54	NA	NA	NA	NA	[43]
	NA	NA	NA	NA	1.13–1.23	[44]
	1.02–1.10	NA	NA	NA	NA	[45]
	NA	NA	NA	NA	0.17	[46]
Eleutheroside E	0.19–23.62	NA	NA	NA	NA	[42]
	NA	NA	NA	NA	2.75–2.99	[44]
	2.53–2.77	NA	NA	NA	NA	[45]
	NA	NA	NA	NA	0.05	[46]
	NA	NA	NA	NA	0.28–0.74	[47]
Liriodendrin	0.07–0.22	NA	NA	NA	NA	[43]
**Coumarins**						
Isofraxidin	trace–2.00	NA	NA	NA	NA	[42]
	NA	NA	1.56 (in rhizome)	NA	NA	[48]
	NA	NA	NA	NA	0.02	[46]
	0.01–0.03	NA	NA	NA	NA	[43]
**Phenolic compounds and their derivatives**						
Chlorogenic acid	0.37–17.64	NA	NA	NA	NA	[42]
	NA	14.74–14.98	4.81–5.21	0.56–0.58	5.90–6.14	[29]
	NA	NA	NA	NA	0.46 *	[46]
	NA	0.71–19.33	NA	NA	NA	[49]
	0.09–0.65	NA	NA	NA	NA	[43]
Caffeic acid	trace–0.31	NA	NA	NA	NA	[42]
	NA	3.07–4.01	0.07–0.13	ND	ND	[29]
	0.03–0.08	NA	NA	NA	NA	[43]
Protocatechuic acid	NA	NA	NA	NA	2.98 *	[46]
	NA	0.33–1.77	NA	NA	NA	[49]
	3.17–23.12 *	NA	NA	NA	NA	[43]
Mono-hydroxycinnamoylquinic acid derivatives	NA	0.55–0.57	0.31–0.33 (shoots)	NA	0.55–0.57	[28]
Di-hydroxycinnamoylquinic acid derivatives	NA	5.08–5.14	2.97–3.07 (shoots)	1.55–1.59	0.04–0.06	[28]
Hydroxycinnamoylshikimic acid derivatives	NA	0.77–0.79	0.25–0.27	0.02–0.04	ND	[28]
Flavonoid derivatives	NA	4.05–4.11	6.73–6.75 (shoots)	0.80–0.82	<0.01	[28]
Ciwujianoside C4	NA	0.27–36.73	NA	NA	NA	[49]
Methyl 5-O-feruloylquinate	NA	1.37–15.41	NA	NA	NA	[49]
Rutin	NA	0.1–11.11	NA	NA	NA	[49]
Hyperoside	NA	0.63–36.56	NA	NA	NA	[49]
**Saponins**						
Ursolic acid	NA	NA	NA	0.18–0.24	NA	[50]
	NA	NA	NA	NA	0.02	[46]
Saponin P_E_	0.89–14.71	NA	NA	NA	NA	[49]
3-O-α-L-rhamnopyranosyl-(1→2)-α-L-arabinopyranoside-29-hydroxy oleanolic acid	NA	0.46–16.20	NA	NA	NA	[49]
3-O-β-D-glucopyranosyl-(1→2)-α-L-arabinopyranoside-29-hydroxy oleanolic acid	NA	0.31–11.86	NA	NA	NA	[49]

* Value in µg/g; NA—not evaluated; ND—not detected, <LOD—below the limit of detection.

### 4.4. Other Phenolic Compounds

Flavonoids are among the most important active compounds in ES. They mainly occur in plant material in both free and glycosidic forms [14] and are mostly located in the cell wall [51]. Their content is affected by various factors, including the harvest period, area, processing method, as well as storage time and conditions [27,31]. Furthermore, the methodological conditions of the methods used to extract, purify, and detect phenolic compounds from plant material influence the quantitative data and make it difficult to compare the results obtained by different research groups. The highest concentrations of phenolic compounds from the group of phenolic acids (chlorogenic acid and caffeic acid) and flavonoids (rutin and quercetin, as well as hyperoside and orientin) occur in the fruits and leaves of ES [14,50]. Chlorogenic acid, p-hydroxybenzoic acid, vanillic acid, syringic acid, p-coumaric acid, caffeic acid, and ferulic acid have been reported in the roots [52]. Kwon et al. reported that the highest content of phenolic acids was found in the leaves of ES (646.56 mg/100 g DW), followed by the shoots (359.19 mg/100 g DW), fruits (167.18 mg/100 g DW), and stems (15.82 mg/100 g DW) [28]. The same descending order was appropriate for flavonoid content (Table 2).

Some new bioactive compounds have been identified in plants. Chen et al. isolated a new aurone, (2Z)-2-[(4′-hydroxy-3′-methoxyphenyl)methylene]-6-methoxy-7-prenyl-3(2H)-benzofurane, from the ethyl acetate-soluble extract of the stems of ES [53]. Furthermore, compounds belonging to caffeoylquinic acids (such as 1-O-caffeoylquinic acid, neochlorogenic acid, chlorogenic acid, and cryptochlorogenic acid), dicaffeoylquinic acids (1,4-di-O-caffeoylquinic acid, 3,4-di-O-caffeoylquinic acid, 1,5-di-O-caffeoylquinic acid, 3,5-di-O-caffeoylquinic acid, and 4,5-di-O-caffeoylquinic acid), and malonyl-dicaffeoylquinic acids (such as 3-O-malonyl-1,5-di-O-caffeoylquinic acid, 3-O-malonyl-1-O-caffeoyl-5-cis-O-caffeoylquinic acid, and 4-O-malonyl-1,5-di-O-caffeoylquinic acid) were identified in the plant extracts [28].

**Table 2:** Comparison of total contents of different groups of bioactive constituents in ES.

Type of Material	TPC	TFC	TAC	Total Saponin	Total Polysaccharide	References
**Stem**	23.50 ± 0.42 mg GAE/g DW	11.49 ± 1.49 mg RE/g DW	2.88 ± 0.28 mg CE/g DW	NA	NA	[29]
**Root**	44.00 ± 0.14 mg GAE/g DW	36.49 ± 0.37 mg CE/g DW	7.61 ± 0.56 mg CE/g DW	NA	NA	[28]
**Leaf**	NA	NA	NA	25.72 ± 0.11 mg/g DW	NA	[28]
	56.08 ± 0.47 mg GAE/g DW	41.23 ± 1.98 mg RE/g DW	8.75 ± 1.02 mg CE/g DW	NA	NA	[29]
**Fruit**	229.83 ± 9.34 mg GAE/g (methanolic extract)	62.25 ± 7.12 mg QE/g (methanolic extract)	NA	NA	NA	[50]
	25.70 ± 0.69 mg GAE/g DW	16.44 ± 0.99 mg RE/g DW	10.86 ± 0.49 mg CE/g DW	NA	NA	[29]
	NA	NA	NA	3.57 mg/g	NA	[31]
**Shoot**	NA	NA	NA	35.77 ± 0.24 mg/g DW	NA	[28]
**Plant**	NA	NA	NA	NA	35.45 ± 0.39 mg/g	[54]
	24.93 ± 0.23 mg GAE/g (in 1-butanol phase)	61.0 ± 0.34 mg RE/g (in 1-butanol phase)	NA	17.80 ± 0.59 mg ginsenosides/g (in 1-butanol phase)	20.04 ± 0.78 mg glucose/g (in water phase)	[51]

NA—not evaluated; CE—catechin equivalent; GAE—gallic acid equivalent; QE—quercetin equivalent; RE—rutin equivalent; TAC—total proanthocyanidin content; TFC—total flavonoid content; TPC—total phenolics content.

### 4.5. Volatile Compounds (Essential Oil)

Only a few studies have focused on the quantification of volatile compounds in ES. The total yield of essential oils may reach approximately 2 mg/g in the fruit [36]. In the stems and roots of ES, iso-caryophyllene and caryophyllene oxide were the predominant essential oil constituents [27,55], accounting for about 10% and 16.4% of the total volatile oil amount, respectively. The most abundant essential oil ingredients in ES fruits were *trans*-caryophyllene (21.7%), humulene (7.4%), bicyclogermacrene (6.0%), (+)spathulenol (4.5%), germacrene-D (3.2%), *tau*-muurolol (2.5%), and *delta*-cadinene (2.3%) [50]. Other volatile compounds that might be found in different parts of the plant, but in lower amounts, are (E,E)-2,4-decadienal, α-pinene, β-farnesene, humulene oxide, humulene, tetradecanal, *p*-cymene, 2-*n*-pentylfuran, *n*-octadecanol, manoyl oxide, 9,17-octadecadienal, linalool, and *n*-heptaldehyde [27,55].

### 4.6. Saponins

Saponins are triterpenoid or steroidal glycosides containing in their structure a lipophilic group (known as ES aglycone or sapogenin) attached to hydrophilic sugars [56]. This class of compounds occurs in different parts of the plant. Ciwujianosides A1, A2, A3, A4, B, C1, C2, C3, C4, D1, D2, D3, and E [27] and acanthopanaxosides A, B, and C [57] were isolated from ES. The plant also includes aglycones, such as mesembraynthemoidic acid, echinocystic acid, hederagenin, serratagenic acid, 11-deoxy-anhydrochiisanogenoic acid, akebonic acid, oleanolic acid, and chiisanogenin [28]. Furthermore, the presence of sessiloside, tauroside H1, chiisanoside, and hederasaponin B has been reported in the leaves [57]. The total saponin content might be higher than that of phenolic compounds, and they accumulate predominantly in the shoots, leaves, and fruits of ES and could not be detected in stems [28].

Saponins have been identified by different research groups. In 2000, Park et al. reported four triterpene glycosides in the leaves: inermoside, 1-deoxychiisanoside, 24-hydroxychiisanoside, and 11-deoxyisochiisanoside [58]. In 2021, Zhang et al. identified new sesquiterpenoids (acasenterpene A–E) [59], while Liu et al. presented five new oleanane-type triterpenoid saponins (acasentrioid A, acasentrioid B, acasentrioid C, acasentrioid D, and acasentrioid E) isolated from ES fruits [60].

### 4.7. Polysaccharides and Glycoproteins

Polysaccharides are widely available in ES [61]. The content of alkaline and water-soluble polysaccharidesin in ES was estimated at 2–8% and 2.3–5.7%, respectively [62]. In 2020, two new pectin polysaccharides, ASP-B2 (M*w*, molecular weight, 5.32 kDa) and ASP-B3 (M*w*, 30.51 kDa), were obtained from ES [61]. These water-soluble polysaccharides showed strong DPPH and hydroxyl radical-scavenging activities in vitro. In 2024, scientists from China isolated and purified a new water-soluble polysaccharide from ES root and structurally characterized it as CQ-1. This polysaccharide exhibited antioxidant and prebiotic activities in vitro [63].

Glycoproteins isolated from ES have beneficial health properties. For example, glycoproteins (M*w*, 30 kDa; N-terminal sequence: NH_2_-Val-Ala-Tyr-Pro-Trp-Ala-Gly-Phe-Ala-Leu-Ser-Leu-Glx-Pro-Pro-Ala-Gly-Trp-) isolated from the stem bark of ES showed DPPH scavenging activity, inhibited lipid peroxidation, and protected against acute and chronic alcohol-induced hepatotoxicity [64].

### 4.8. Recent Developments in Terms of Evaluation of ES Phytochemical Composition

**Figure 4:**
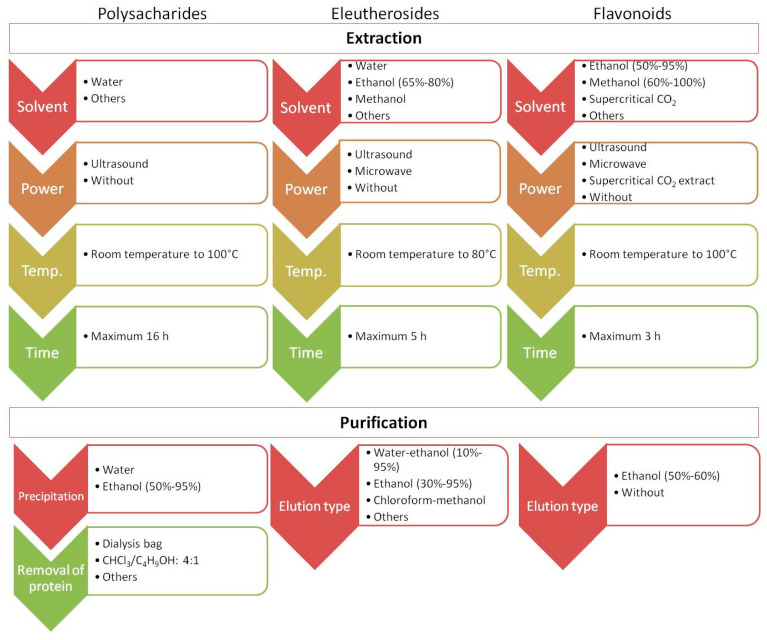
The juxtaposition of extraction parameters and purification conditions during the determination of polysaccharides, eleutherosides, and flavonoids.

Recently published studies have focused on developing new and more effective approaches to isolate and identify target analytes from ES. Importantly, one method is not sufficient to obtain information on the global quantitative and qualitative composition of ES. For example, the content of polysaccharides, which are high-molecular-weight natural polymers, in plant materials is usually estimated by colorimetry (e.g., the phenol–sulfuric acid and anthrone–sulfuric acid methods). However, before analysis, the plant material underwent extraction (e.g., using hot water or under alkaline conditions) and purification. Methodological issues in terms of extraction, purification, and structural characteristics of polysaccharides in ES were discussed in a recent review [31,62] and are briefly summarized in Figure 4. Comprehensive profiling of small constituents of ES (such as ES metabolites) may be performed using approaches based on liquid or gas chromatography separation followed by UV or mass spectrometric detection. Phenolic acids show maximum UV absorption at approximately 320 nm, while flavanones and flavanols show maximum absorption at 280 nm and 350 nm, respectively. Unlike phenolic compounds, triterpenoid saponins are difficult to analyze using UV spectroscopy, and engagement with another type of detector is necessary [28]. Mass spectrometry (MS) detectors are known for their selectivity, sensitivity, and versatility. Their combination with liquid chromatography (LC-MS) or gas chromatography (GC-MS) allows for the simultaneous detection and determination of numerous major or trace components of ES during a single analytical run. Through non-target metabolomic analysis accompanied by switching the polarity to detect both negative and positive ions generated in the MS ion source, comprehensive profiling of the plant extract is possible as well as identification of more than 1300 analytes [65]. Thus far, the LC-MS approach has allowed the identification of a wide range of phenolic compounds (50 analytes) and triterpenoid saponins (82 analytes) in different parts of the plant [28]. The combination of both GC-MS and ultra-high-performance liquid chromatography–quadrupole time-of-flight mass spectrometry (UHPLC-QTOF-MS/MS) allowed the separation and identification of compounds of ES fruits, receiving unique fingerprints from plants and relative area percentage of volatile and fatty oil components [66].

Currently, there is a great need to find non-toxic, easy-to-dispose, and effective solvents with high extraction yields for extraction purposes. It is also important to consider the energy usage. To address this, Pan et al. presented a new method based on microwave distillation and extraction using deep eutectic solvents (DESs) for multiple analytes prepared from ES fruits. This method allowed for the successful extraction of eleutheroside B, eleutheroside E, isofraxidin, anthocyanins, and essential oils from ES fruit [36]. Recently, DESs have gained increased interest because of their indisputable advantages, including the ability to extract components with different polarities, ease of preparation, and high extraction efficiency. DESs are known as environmentally friendly solvents for ES, which enhances their attractiveness. The combination of DESs and microwave technology effectively increases mass transfer and extraction efficiency compared to traditional approaches [36]. Microwave-assisted extraction also has multiple benefits, such as high extraction effectiveness, short extraction times, minimal energy usage, and reduced solvent amounts. The proposed approach has some limitations (the suppression of extraction due to the excessive viscosity of DESs, as well as uneven material heating or even gelatinization during microwave extraction) [36]. However, there are some possibilities for modifying these approaches to overcome these limitations. Thus, the potential is significant. DESs can also be utilized with ultrasonic extraction, for example, for eleutheroside E and B isolation [44].

Liu et al. presented a new approach for the extraction of flavonoids from ES [51]. They proposed enzyme-assisted extraction under the following conditions: a 3:2 ratio of cellulase to pectinase, enzyme mixture (6960 U/g), enzyme treatment time of 59.80 min; temperature of 53.70 °C, and pH value of 6.05. This type of extraction is highly effective and sustainable and is suitable for isolating bioactive compounds from materials with a high cellulose content [51]. They also found that among all tested solvents (1-butanol, chloroform, ethyl acetate, water, and petroleum ether) for extract purification, 1-butanol was the most suitable for enriching flavonoids from the extract of ES.

## 5. Specific Scientific Research Confirming the Biological Activity Profiles

The pharmacological properties of ES have been studied extensively [14]. The Chinese Pharmacopoeia states that it is a traditional Chinese drug that is frequently administered to strengthen the spleen, tonify the kidney, calm the mind, and ‘nourish qi’ [67]. It mostly affects the central nervous system and the cardio-cerebrovascular system. Additionally, ES exhibitsanticancer, antioxidant, anti-inflammatory, anti-bacterial, anti-gout, anti-hyperglycemic, anti-leishmanicidal, anti-hepatitis, and antipyretic effects, as well as choleretic, hemostatic, immunostimulatory, hypocholesterolemic, and radioprotectant effects [68,69,70,71]. In vivo and in vitro studies on the biological activities of ESare shown in Table S1 (see Supplementary Materials).

### 5.1. Adaptogenic Activity

In ES, the adaptogenic effect is particularly important. It is associated with an increase in the body’s ability to cope with physical exertion, stress, and mental strain [1]. ES also has a positive effect on the physical endurance of the body [25]. Clinical studies have been conducted on people who practice sports. It has been shown that preparations containing ESsubstantially improve circulatory system function during exercise, which improves the efficiency of the body. They also increase the amount of simple fat metabolized. The subjects reported that they felt more confident and stronger during exercise with no side effects [72]. The improvement in physical fitness and decreased fatigue werealso confirmed in another study, that is, fitness tests on a group of swimmers. People consuming ES could swim significantly longer before they experienced significant fatigue, which prevented further activity [73].

The adaptogenic effect of preparations containing ES raw material is also associated with its neuroprotective effect. ES extracts can inhibit neurodegenerative processes, which form the basis of many neurological diseases. In vitro studies on microglia and hippocampal cells have shown that the compounds contained in ES inhibit inflammation-induced neuronal apoptosis. Additionally, they exhibit antioxidant properties [74]. These properties were confirmed in studies on rats in which cerebral ischemia was artificially induced. Animals administered the bark extract of ES showed a smallerdecline in task-solving performance than the control group [75].

### 5.2. Antidiabetic Activity

Currently, increasing attention is being paid to the hypoglycemic effects of ES. These effects are attributed to eleutherosides B and E. Both compounds have been studied in animals suffering from type II diabetes. It has been shown that they significantly reduce the concentration of glucose in the plasma compared to the control sample. It has also been found that eleutheroside E can protect the β cells of the pancreas and improve the response of cells to insulin. However, these results have not yet been confirmed in clinical trials [34,35]. According to previous research, ES may have an impact on the immunological and endocrine systems, which could lower blood sugar levels in animal models of diabetes. Watanabe et al. [76] showed that ES extract administered to db/db mice over a three-day period enhanced glucose tolerance, inhibited small intestinal glucosidase activity, and induced glucose regulation comparable to a pharmaceutical drug. In a separate study, Zhou et al. [77] identified phenolic acids (such as 1,5-dicaffeine quinic acid and 4,5-dicaffeine quinic acid), flavonoids, and 1,4-dicaffeine in ES using UF-LC/MS and ESI-MS. These substances showed α-glucosidase inhibitory effects, indicating that ES could be used to create α-glucosidase inhibitors, which are known to be effective therapies for diabetes. Lignan compounds derived from the soluble ethyl acetate fraction also demonstrated potential as inhibitors of protein tyrosine phosphatase 1B (PTP1B), which may be used to treat type 2 diabetes [78]. All these trials suggest that ES may be useful in preventing type II diabetes and controlling postprandial hyperglycemia.

### 5.3. Neuroprotective Activity

The use of ES in the treatment of neurological conditions has recently gained attention. There are many reported neuroprotective compounds in ES, like isofraxidin, eleutherosides B and E, as well as sesamin. Based on its neuroprotective effects, ES has demonstrated potential for enhancing memory and cognitive function, especially in diseases such as Parkinson’s and Alzheimer’s disease [21,79]. For example, Eleutheroside B is effective in protecting against neuronal atrophy associated with Alzheimer’s disease [80]. In contrast, saponin extracts from ES leaves have been associated with improved memory and learning capabilities in rats, suggesting a broader cognitive-enhancing effect [81]. It also has neuroprotective effects against cerebral ischemia, depression, and fatigue. The neuroprotective mechanism is linked to the activation of multiple signaling pathways, including the PI3K-Akt pathway, which is crucial for neuronal survival and growth. Fujikawa et al. [82] studied noradrenaline (NA) and dopamine (DA) levels in rat brains (whole or block tissues) through single- or two-week daily administration of ES harms (ASH) extract. A single dose of 500 mg/kg significantly increased DA levels in the pons and striatum, while lowering them in the substantia nigra and the posterior hypothalamus. Considering the 2-week administration group using the same extract dose, a significant increase in DA levels in the striatum and anterior hypothalamus, as well as a decrease in the substantia nigra and posterior hypothalamus, was recorded. ES elevated the accumulation of 3,4-dihydroxyphenylacetic acid (DOPAC) and 3-methoxytyramine (3-MT) in the anterior hypothalamus, whereas ASH increased all DA metabolites tested solely in the striatum. The dosing for two weeks also raised 3-MT levels in the occipital brain and posterior hypothalamus, as well as DOPAC levels in the frontal cortex and posterior hypothalamus. According to these findings, ASH’s effects on NA and DA may help avoid some stress-related illnesses as well as movement abnormalities commonly associated with Parkinson’s disease.

Additionally, new research indicates that phenylpropanoid components isolated from ES have strong potential for creating Alzheimer’s disease treatment plans [83]. Phenylpropanoid improved cognitive function in SAMP8 mice and reduced Alzheimer’s disease symptoms, according to in vivo research. This substance reduced intracellular ROS levels in vitro. Mechanistically, cellular thermal shift assay analysis verified the binding affinity between phenylpropanoid and Mst1, demonstrating that phenylpropanoid reduced ROS levels and alleviated oxidative stress via modulating the Mst1 signaling pathway.

### 5.4. Anticancer Activity

Extracts of ES contain various bioactive compounds, including polysaccharides, sesamin, and isofraxidin, which exhibit antitumor potential. Isolated from the stem bark, sesamin could stimulate the apoptotic process and prevent the development of KATO III cells associated with human stomach cancer. Evidence of its activity includes the presence of oligonucleosomal-sized DNA fragments and apoptotic bodies in cells treated with ES [84]. Sesamin demonstrated a dose-dependent effect, reducing cell viability while enhancing LDH release and apoptosis (as indicated by the TUNEL assay). This implies that sesamin could be utilized as a dietary supplement to prevent breast cancer by restricting tumor cell proliferation and altering apoptotic signaling pathways [85].

Using two human breast cancer cell lines (MCF-7 and MDA-MB-231), Hwang et al. [86] examined the anticancer properties of an ES fruit water extract. Both types of breast cancer cells’ proliferation wasinhibited by ES fruit treatment, the impact grew stronger with increasing dosage. Additionally, ES enhanced the mRNA expression of pro-apoptotic genes while decreasing that of the apoptotic suppressor gene Bcl-xL. In both cell types, ES fruit raised the mRNA expression of RIP-1 and p21. In the MCF-7 cell line, ES fruit also reduced survivin’s mRNA expression. Apoptotic cell death is one way that ES fruit demonstrates anticancer action.

According to Kou et al. [87], EGFR, MAPK3, ICAM1, and CTSK are important mediators of apoptosis induced by ES fruit, as shown by multi-layered transcriptomic, metabolomic, and in vitro validation data. Its primary bioactive compounds, nitro-linoleic acid and calycanthoside, can either directly or indirectly affect the transcription factors POU2F3, FOXS1, and TGIF2LY’s binding affinities for the promoters of EGFR, MAPK3, ICAM1, and CTSK. These findings are supported by additional correlation analyses and molecular docking studies. However, the authors assert that comprehensive mechanisms are necessary to further validate monomer compounds and associated molecular biology experiments.

Other components of the plant have also shown promise in the treatment of metastases. When the soluble protein layer (GF-AS) of ES was administered to mice with colon26-M3.1 cancer cells before pre-inoculation, fewer cells spread to the lungs than in the control group [88]. This component operates in a dose-dependent manner in addition to preventing metastasis. This function may be caused by cell ligand–receptor interactions. When it came to preventing tumor metastases in mice, glycoproteins (EN-SP) were more effective than GF-AS, which required 5–50 μg/20 g and only 1–5 μg/mouse [88]. At a higher dose of 100–500 μg/mouse, GF100, another extract, was effective in preventing colon26-M3.1 sarcoma cells from spreading to the lungs [71]. When these three solutions are compared, EN-SP was the most successful in preventing metastases. It is also probably a major ingredient in GF-AS and GF-100 extracts.

The growth of SW982 human synovial sarcoma cells is significantly inhibited by isofraxidin, with inhibition reaching 36.2% at 150 µM, but SR and syringin did not result in such notable growth reductions under the same conditions [89]. Additional research on isofraxidin has demonstrated that it inhibits hepatoma cell invasion in vitro [90]. The effects of Siberian root tincture on radiation-induced cancer have also been the subject of somegeneralin vivo studies [91]. The above authors examined the overall macroscopic effects of tincture and compared them with those of α-difluoromethylornithine(DFMO), a well-known synthetic anticancer medication. Only 58.7% of mice treated with DFMO and 62.1% of mice treated with Siberian root tincture developed tumors compared to 79.6% of untreated irradiated control mice. Both were much lower than the control, indicating that the tincture had an antitumor effect that was not as strongas that of DFMO, but still much stronger than that of the control. This comparative analysis shows how important it is to examine how ES affects cancer.

Another antitumor component of ES iseleutheroside B, which is an effective treatment for breast cancer. According to Lee et al. [92], it can cleave and activate caspases 3/9 and poly (ADP-ribose) polymerase (PARP), downregulate the expression of X-linked inhibitor of apoptosis protein (XIAP), block the cell cycle in the G2/M phase, and induce oxidative stress to inhibit breast cancer proliferation. With a half-maximal inhibitory concentration (IC_50_) of 160 mg/mL, polysaccharides isolated from ES roots can prevent the proliferation and metastasis of human non-small-cell lung cancer NCI-H520 cells by down-regulating Wnt/β-catenin signaling components, leading to reduced nuclear β-catenin and cyclin D1 levels [93]. Although numerous studies have documented the antitumor effects of ES, how it influences apoptosis and epigenetic regulation is still unknown.

### 5.5. Antioxidative Activity

Flavonoids from ES are the primary bioactive components with antioxidant properties. Su et al. [94] showed that ES flavonoids significantly increased antioxidant activity in mice with DSS-induced colitis and RAW 264.7 cells triggered by H_2_O_2_. The results of their investigation demonstrated that ES flavonoids can prevent colitis by enhancing antioxidant activity in mice and preserving the normal physiological function of the intestinal tract, in addition to protecting macrophages under oxidative stress by boosting the activity of antioxidant enzymes within the cell and activating the Nrf2/Keap1/HO-1 signaling pathway.

Following oral administration at doses of 50 mg/kg, 100 mg/kg, and 150 mg/kg of body weight daily, ES polysaccharides significantly increased SOD and catalase activities, decreased blood glucose levels, and dose-dependently lowered levels of lipid hydroperoxides and lipid peroxidation markers in alloxan-induced diabetic mice [95]. Because of its antioxidative properties, ES extract is a natural antioxidant food additive, in addition to being a nutraceutical. Eleutheroside E1, the isolated component, exhibits potent anti-2,2-diphenyl-1-picryl-hydrazyl-hydrate radical (DPPH) action compared to the raw extract (half maximal effective concentration of 37.03 μg/mL; 63 mM). Its primary antioxidant action is based on the complexation of aryl radicals with DPPH molecules. The methoxy groups in the aromatic rings of aryl radicals can stabilize them, enhancing their antioxidant properties [96]. Syringin, eleutheroside E, and ES flavones and polysaccharides were examined for their antioxidant properties using DPPH and 2,2-azino-bis(3-ethylbenzothiazoline-6-sulfonic acid) tests. Compared to syringin and eleutherosideE, ES flavones and polysaccharides exhibited superior radical-scavenging efficacy at 5 mg/mL. Additionally, the antioxidant activity was investigatedin vivo. Mice were exposed to matrix-assisted laser desorption/ionization and heavy-ion radiation. Heat shock protein 90B1, disulfide isomerase polysaccharides, and flavonoids were found to be efficient in protecting mice from tissue oxidative damage caused by heavy ion radiation. Understanding the exact process by which these proteins are downregulated will aid in the regulation of ES [97].

The antioxidant activity of ES stems was also demonstrated in CCI_4_-intoxicated rats [98]. Lignan-eluteroside B was found to have a moderate effect on DPPH in terms of free radical scavenging. These findings imply that ES stems have hepatoprotective and antioxidant properties in rats. In vitro tests of the antioxidant effects of extracts of various parts of ES revealed that the fruit extract had the strongest antioxidant effects based on ABTS and FRAP assays, as well as the highest reducing power and ORAC. The leaf extract of ES had the strongest effects based on DPPH radical scavenging ability [29]. Additionally, ES leaves contain more caffeic acid and chlorogenic acid than other plant components do. According to Su et al. [99], there is a substantial correlation between H_2_O_2_-induced oxidative stress in PC12 cells and many metabolites found in different parts of ES. Furthermore, these investigations showed that ES roots had a higher concentration of antioxidant components than seeds and leaves.

### 5.6. Immunomodulatory Activity

Numerous studies on various ES components, such as eleutheroside B (syringin), (+)-syringaresinol-di-O-β-D-glucoside, and isofraxidin, have highlighted their immunomodulatory properties [89,100]. This activity has been confirmed in clinical studies and is conditioned by the presence of specific polysaccharides and glycoproteins (EN-SP) in the raw material. Together, these factors lead to an increase in the number of leukocytes in the blood and stimulate antibody production. Eleutheroside E, which has immunomodulatory properties, contributes particularly to strengthening the immunity of patients [101]. In addition, studies on mice with artificially induced sepsis showed increased survival of animals administered an extract from the underground parts of ES. Interestingly, a dose of 400 mg/kg of body weight allowed 100% of mice from this sample to survive for up to 72 h. In comparison, among the mice that were not administered the extract, death occurred within 10 h [102].

As stated by Yamazaki et al. [89], these substances dramatically reduce the production of IL-1β and IL-6 by immune cells. Of these, (+)-syringaresinol-di-O-β-D-glucoside was the most effective in suppressing IL-6 at 50 μM, whereas isofraxidin required 450 μM to have the same effect. This implies that while all of the drugs might function via comparable mechanisms, (+)-syringaresinol-di-O-β-D-glucoside exhibited a higher inhibitory effect on both cytokines, which can be attributed to its unique composition. Additionally, syringin exhibits immunomodulatory properties against CD8+ T-cells. Syringin suppressed the growth of these cells when a mitogen (Con A) was present, but did not affect the production of NO or the growth of CD4+ T cells. Further research is required to clarify how syringin inhibits the proliferation of cytotoxic T-cells and to understand how the components of ESaffect CD8+ and CD4+ T cells differently. Syringin also greatly decreased the amount of TNF-α produced by murine macrophages in invitro experimental setups [100]. A plausible mechanism of action could be that syringin interacts with receptors on the surface of macrophages or acts on TNF-α after it is produced, changing its composition and preventing detection. According to a study by Schmolz et al. [103], an ethanolic extract of ES significantly inhibited the release of IL-4, IL-5, and IL-12, and affected cytokine synthesis in activated whole blood cultures of ten healthy people. This suggested that the preparation had an immunomodulatory effect. Additionally, the ethanol extract of ES was able to stimulate and boost interleukin-1 and interleukin-6 production in vitro, but not interleukin-2 [104]. The generation of important cytokines and markers for immunological activation, as well as the activation and proliferation of immunocompetent cells, is affected by ESin vitro. When evaluated on cultured whole blood cells, a standardized fixed mixture of extracts from *Andrographis paniculata* and ES increased the production of TNF-α and beta-MG more than either single pure extract [105].

Using in vitro experiments, Han et al. [106] examined the mechanism underlying the immunomodulatory effect of a polysaccharide fraction extracted from an ES cell culture. They discovered that ES enhanced B cell proliferation and B cell production of polyclonal IgM antibodies in a dose-dependent manner. Additionally, ES activated murine peritoneal macrophages, evidenced by the increase in cytokine mRNA and protein expression, including TNF-α, IL-1β, and IL-6. Furthermore, ES boosted nitric oxide production and expression of the inducible nitric oxide synthase gene. ES activates B cells and macrophages through the Toll-like receptor signaling pathway. This conclusion was supported by experiments showing that pretreatment of cells with antibodies targeting TLR-4 and TLR-2 significantly attenuated ES-induced cellular responses. In contrast, it was demonstrated that ES did not exert stimulatory effects on T cells. The ES polysaccharide was found to have an immunomodulatory effect on chickens that were immunocompromised by cyclophosphamide [107]. Both high and low doses of ES polysaccharides were able to considerably boost lymphocyte proliferation at most time periods, raise body weight, antibody titers, and percentages of CD41 and CD81 T cells, and increase interferon-gamma and IL-2 levels in chickens treated with cyclophosphamide compared to chickens in the cyclophosphamide control group. According to these findings, ES polysaccharides may be a novel type of immune adjuvant that can enhance vaccination in both healthy and immunocompromised chickens and resistcyclophosphamide-induced immunosuppression.

A recent research by Wang et al. [108] showed that the polysaccharide component from ES leaves exhibitsstrong immunomodulatory properties and may be a natural immune enhancer. In in vitro studies, ES significantly activated macrophages by increasing levels of NO, iNOS, TNF-α, IL-1β, and IL-6. iNOS, TNF-α, and IL-6 expression depended on the TLR4 receptor and IL-1β mainly on TLR2; TNF-α regulated both receptors. Bioinformatics analyses indicated a key role for MAPK and NF-κB pathways. Molecular docking, on the other hand, confirmed that ES binds to TLR4 and TLR2, more strongly to TLR4, forming stable complexes through hydrogen bonds and hydrophobic interactions.

According to many of the studies presented above, active ingredients from ES, especially syringin, isofraxidin, and polysaccharides, show promise as having anticancer and immunostimulatory effects. Direct tumor inhibition and immune activation have both been shown in vitro; if these two effects are verified simultaneously in animal models (and eventually in humans), ES may prove to be a successful anticancer treatment. However, more clinical and in vivo studies are still required.

### 5.7. Cardiovascular and Cerebrovascular Activities

Numerous studies have examined the cardiovascular effects of ES, emphasizing its potential advantages in improving endurance and reducing stress reactions. These data point to the potential benefits of ES supplementation in the metabolic and cardiovascular systems. Kuo et al. [72] stated that an eight-week supplementation of ES extract significantly increased the maximum heart rate by 4% (*p* < 0.05) during exercise, suggesting improved cardiovascular function.

Facchinetti et al. [109] used the Stroop Color–Word (Stroop CW) test as a challenge stressor before and after treatment with a placebo or ES extract. The extract significantly reduced cardiovascular responses to stress, evidenced by a 40% decrease in heart rate reactivity and a 60% reduction in systolic blood pressure in females during the Stroop CW test compared to placebo. In another study, ES was shown to have a protective effect against cardiac ischemia-reperfusion damage in rats [110]. Rats were administered ES saponins at doses of 25, 50, and 100 mg/kg 30 min after coronary occlusion. The results showed a significant decrease in myocardial infarct size, a significant increase in plasma prostacyclin and thromboxane A2 levels, a decline in serum creatine phosphokinase and lactate dehydrogenase activity, and a decline in myocardial free fatty acid content. Additionally, serum lipid peroxidation levels decreased, whereas superoxide dismutase and glutathione peroxidase activities significantly increased.

### 5.8. Anti-Inflammatory Activity

Experimental studies have shown that ES exhibits anti-inflammatory properties both in vitro and in vivo. By suppressing aberrant autophagy in pancreatic acinar cells, Wang et al. [111] found that oral administration of ES (3.5 mg/100 g) showed therapeutic potential against severe acute pancreatitis caused by sodium taurocholate in rats. In a subsequent study, Wang et al. [112] investigated the anti-inflammatory effects of ES flavonoids on LPS-induced intestinal inflammation using in vivo studies. Through the prevention of intestinal damage, reduction of inflammation, maintenance of the intestinal barrier, and control of gut microbiota balance, ES flavonoids produced an anti-inflammatory effect in mice. The proposed anti-inflammatory mechanism may occur through the TLR4/NF-κB signaling pathway in ES flavonoids [112]. Similarly, by altering the nuclear factor κB(NF-κB) pathway, Fei et al. [113] observed that ES decreased the levels of IL-6 and TNF-α in the lung tissues of a mouse model of acute lung damage. Furthermore, focusing on the NF-κB/MLCK pathway, Han et al. [114] showed that ES polysaccharides may lessen sepsis induced by lipopolysaccharide (LPS). Furthermore, by improving myocardial calcium channel activity, Guan et al. [115] discovered that ES flavones can mitigate myocardial ischemic injury in rats. Polysaccharides isolated from ES could alleviate LPS-induced intestinal inflammation in piglets by regulating gut microbiota and hyodeoxycholic acid levels [116]. These latest studies demonstrate the therapeutic potential of hyodeoxycholic acid in reducing intestinal inflammation in piglets and may offer compelling evidence for ES polysaccharide as a feed supplement to alleviate piglet diarrhea.

### 5.9. Antiulcer Activity

Fujikawa et al. studied the pharmacological effects of ES stem bark on stress-induced stomach ulcers and discovered that a single oral dose of an aqueous extract had no preventive effect [117]. However, after oral administration of the extract for two weeks, a dose-dependent protective effect was observed. Additionally, an n-butanol extract administered orally for two weeks demonstrated a clear 61.1% inhibition of stomach ulcers. This study showed that syringaresinol di-O-β-d-glucoside and chlorogenic acid may play a role in the ability of ES stem bark to prevent stomach ulcers.

## 6. Forms of Administration, Dosage, and Toxicity

Due to the widespread popularity and long history of the use of *Eleutherococcus senticosus*, this plant is incorporated into a wide variety of pharmaceutical formulations and dietary supplements worldwide. The most common forms include dried powdered rhizomes, aqueous and hydroalcoholic extracts, tinctures, capsules, and tablets. Ethanol and ethanol–water extracts are typically prepared in a ratio of 10:1, ensuring a standardized concentration of active compounds such as eleutherosides, which are considered pharmacologically relevant constituents [24]. In traditional medicine, particularly in East Asia and Russia, ES is also administered as teas or infusions prepared from the dried aerial parts of the plant, a form still commonly used in contemporary herbal practice [2]. The WHO monograph recommends a daily intake of 2–3 g of the raw dried material, which can be consumed as part of a decoction or incorporated into other preparations [9]. Various clinical studies have explored effective dosages for specific outcomes. For instance, an 8-week study on physically active individuals demonstrated that 800 mg/day (administered as 100 mg capsules, taken multiple times daily) significantly improved physical performance and endurance [72]. Other trials have employed daily doses ranging from 200 to 500 mg of standardized extract, reporting benefits such as improved stress resistance, cognitive performance, and immune modulation. While *E. senticosus* is generally considered safe and well tolerated, its toxicity profile remains only partially elucidated. Mild side effects have been observed, including drowsiness, which may be linked to the plant’s hypoglycemic properties. This effect was reversible with sugar intake, suggesting a functional relationship with blood glucose levels. Other infrequent adverse effects include insomnia, arrhythmias, and headaches; these were primarily reported in studies from the former USSR conducted several decades ago [2], which lack contemporary methodological rigor [10]. According to the European Medicines Agency (EMA), adverse effects associated with ES are rare and poorly documented, and there is a notable lack of long-term safety studies [10]. Moreover, the safety of ES in sensitive populations such as pregnant and breastfeeding women remains under-researched. Historical data suggest a lack of teratogenic effects, and no complications were observed in early clinical evaluations involving pregnant women. Nonetheless, the WHO recommends avoiding use during pregnancy and in individuals with hypertension due to potential interactions with cardiovascular homeostasis [9]. Interestingly, more recent sources have suggested that ES may possess mild antihypertensive properties, though the evidence remains inconclusive and is often confounded by methodological variability. Until more robust data emerge, a cautious approach is advised when recommending SG for individuals with cardiovascular conditions or those on antihypertensive therapy.

An important consideration in the clinical use of *E. senticosus* is its potential to interact with concurrently administered medications. Pharmacokinetic studies have demonstrated that ES preparations may influence the bioavailability and plasma concentrations of certain orally administered drugs. Notably, an interaction with digoxin has been documented, wherein co-administration resulted in a significant increase in serum digoxin levels, likely due to altered gastrointestinal absorption or P-glycoprotein inhibition [118]. Similarly, though less pronounced, interactions have been observed with dexamethasone and diazepam, suggesting modulation of cytochrome P450 enzymes or drug transporters. Additionally, due to its immunomodulatory and adaptogenic activity, ES may theoretically interfere with immunosuppressive treatments or corticosteroids, although this hypothesis has yet to be rigorously evaluated in clinical settings. Individuals on polypharmacy regimens should, therefore, be monitored closely when using supplements containing ES [119].

In summary, although ES exhibits a high safety margin in short-term use, particularly when administered in standardized extract form within recommended dosages, its long-term safety and full interaction profile remain under-investigated. This highlights the need for further controlled clinical trials, especially those assessing its pharmacodynamics, chronic toxicity, and real-world safety in diverse populations [2,9,10,24,26,72,118,119].

## 7. Aspects of Plant Biotechnology Research

### 7.1. Importance of Biotechnology Research

Notably, the recent publication of a chromosomal-scale genome assembly of *E. senticosus* [120] provides a foundational resource for gene discovery, molecular breeding, and metabolic engineering aimed at enhancing the biosynthesis of valuable secondary metabolites in this species.

As previously mentioned, field cultivation of ES is ineffective, which is related to the long stratification process that the seeds must undergo. In addition, the specific environmental requirements of this plant mean that, for example, in China, the raw material obtained from cultivation often does not meet the pharmacopeial requirements for eleutheroside content [121].

Therefore, numerous studies have been conducted on the in vitro cultivation of ES cultures. All types of cultures are well described, including callus cultures on solid media, suspension cultures, and organ cultures in bioreactors. Pilot studies have also been conducted at a semi-industrial scale [122]. Plant biotechnology techniques are used for the production of both active compounds and plants [23].

### 7.2. Micropropagation

ES is a rare species that is protected in some countries and has difficulty in vegetative propagation; therefore, biotechnological methods are frequently used. In vitro culture techniques enable the production of a large number of genetically applied plants with utility profiles in a short time. These techniques use zygotic or somatic embryos to produce explants via micropropagation [20].

Zygotic embryos were obtained from seeds. The isolation of zygotic embryos eliminates the need for long-term stratification. In the case of ES, the use of fresh seeds to obtain zygotic embryos resulted in 56% of germinating explants, and 40% of them produced roots and two normal cotyledons. In the case of seeds stored for one year, only 15.5% germinated; when two-year-old seeds were used, only 1.0% of them germinated. Additional stratification and the addition of plant growth regulators to the medium increased the number of germinating zygotic embryos [20].

Somatic embryos were obtained from the vegetative cells of parent plants. They can develop either through direct embryogenesis (embryos develop from primary explant cells) or indirectly (embryos are produced after prior tissue dedifferentiation). During indirect embryogenesis, calli can be induced and used to obtain suspension cultures for cultivation in bioreactors. The use of special bioreactors allows us to obtain a large number of somatic embryos in a short time [20,22]. In addition, somatic embryos can be used for the production of artificial seeds. From 1 g of embryos, 3.4–6.5 well-developed seedlings with well-developed roots, stems, and leaves were obtained [123].

The most beneficial materials for obtaining somatic embryos are explants from apical buds and fragments of the hypocotyl of seedlings obtained from immature zygotic embryos isolated from seeds. The described in vitro techniques allow for the quick acquisition of large numbers of individual plants, which adapt well to ex vitro conditions, and after a few years, they can become a good source of bioactive compounds, e.g., for industry. Adaptation of plants to ex vitro conditions is an important stage in the production of seedlings to establish plantations. This process depends on many factors such as the type of soil, pH, and photoperiod applied [20].

Somatic embryogenesis in ES explants cannot be induced on Murashige and Skoog (MS) medium without plant growth regulators. 2,4-D was the most efficient compound for somatic embryogenesis induction. In addition, high nitrogen concentration (from NH_4_NO_3_) induced embryogenic callus growth on the medium without regulators [22].

In 2005, a micropropagation system using nodal cutting of stems was established. A comparison of media types (MS, Lloyd and McCown, and Schenk and Hildebrandt media) and the addition of plant growth regulators (various concentrations of auxins, cytokinins, and gibberellic acid (GA_3_)) was conducted. The most beneficial for producing new shoots, shoots elongation, and node formation was the MS medium variant with the addition of 2.0 mg/L benzyl adenine, 0.5 mg/L indolilobutyric acid, and 0.5 mg/L GA_3_. The next step was root induction, for which the most effective method was the use of MS medium without NH_4_NO_3_, with half strength of mineral salts and the addition of 0.5 mg/L 1-naphthaleneacetic acid. Three months after transferring the obtained plants to in vivo conditions, 90.3% of them survived [124].

The problem of germination of long-stored seeds is partially solved by the continuous maintenance of in vitro cultures. Long-term maintenance of embryogenic cells can be laborious, and cultures are susceptible to genetic variability and contamination with pathogens. To overcome these problems, research is being conducted on cryopreservation methods for ES [123]. Cryopreservation allows cells to be stored in liquid nitrogen at −196 °C. This method allows cells to be stored for an unlimited period, provides a continuous source of genetically stable cells, and reduces the risk of contamination [123]. One of the most important factors in a successful cryopreservation system is the cryoprotectant; glycerol and dimethyl sulfoxide are the most commonly used [123].

### 7.3. Production of Secondary Metabolites via Plant In Vitro Culture Methods

Somatic embryos have been proposed for the production of valuable secondary metabolites in ES. To improve biomass growth and increase the production of compounds, scientists have studied various factors influencing culture conditions and the accumulation of bioactive compounds in in vitro cultures. The composition of the media, addition of plant growth regulators, light conditions, and addition of elicitors were tested [21,122].

Maintaining in vitro cultures in bioreactors allows for a significant increase in the amount of biomass and bioactive compound production [122]. ES somatic embryo cultures were optimized in 3 L bioreactors (balloon, bulb, cone, and cylinder bioreactors) on MS medium with various parameters, including aeration volume and inoculum density. The next step was to scaleup the production using 500 L drum- and balloon-type airlift bioreactors. The research was focused on developing a methodology to produce eleutherosides and achieve the highest biomass growth of somatic embryo suspension cultures. The cultures were aerated with volumes of 0.05, 0.1, 0.2, and 0.3 vvm (air volume/L/min) constantly or with 0.05–0.3 vvm increased every 6 days. The tested inocula were 1, 3, 5, 7, and 10 g biomass per 1 L medium. The most favorable conditions for biomass growth of somatic embryos and accumulation of eleutherosides were maintained in balloon-type airlift bioreactors with incrementally increasing aeration volume. The optimal inoculum concentration was determined to be 5 g/L. The highest amount of dry biomass obtained was 11.3 g/L and the highest values of accumulated compounds were eleutheroside B (327 µg/L), eleutheroside E1 (388 µg/L), and eleutheroside E (550 µg/L) (Table 3). It is important to upscale the bioreactor system to a 500 L airlift bioreactor, which did not cause a decrease in biomass growth and eleutheroside production compared to small-scale bioreactors [122]. Root cultures of ES were obtained. Adventitious roots were directly induced from seed-derived plants and maintained in a half-strength MS medium supplemented with 5 mg/L indole-3-butyric acid (IBA) and 0.01 mg/L thidiazurone(TDZ). Cultures were maintained in the dark and transferred to a new medium every five weeks. Root cultures were also successfully transferred to 3 L airlift bioreactors and maintained under the same culture conditions. The inoculum density of adventitious roots was 5 g/L, and the aeration volume was set at 0.1 vvm [125].

The mineral salt content in the culture media is another factor that affects not only the growth of biomass but also the production of metabolites in in vitro cultures. The researchers investigatedthe effects of different concentrations of mineral salts in MS medium (1/4, 1/2, 3/4, 1, and 2 of the basic amount) on the adventitious roots of ES grown in bioreactors. The greatest increase in biomass growth was obtained on media variants with lower concentrations of mineral salts than the standard MS medium amounts, except for the 1/4 variant, where growth was inhibited due to essential mineral deficiency. The most beneficial effect on growth was observed on the medium containing 1/2 mineral salts. Accumulation varied depending on the compounds analyzed. For eleutherosides B and E accumulation, the most beneficial were MS media variants with 1/2 (297 and 756 µg/L, respectively) and 3/4 (284 and 825 µg/L, respectively) salt strengths (Table 3). For the other analyzed compounds, chlorogenic acid, total phenolics, and total flavonoid accumulation decreased with increasing salt strength. Taking into account both the biomass increase and the production of the main compounds of ES (eleutherosides B and E), the optimal addition of mineral salts to MS medium is 1/2 of the basic amount for cultures maintained in 3 L airlift bioreactors for a 5-week growth period [126].

Another variation in the composition of the MS medium in the adventitious roots of ES that was studied was the ammonium-to-nitrate ratio. Seven variants of the NH_4_^+^:NO_3_**^−^** ratio were tested: 0:30, 5:25, 10:20, 15:15, 20:10, 25:5, and 30:0 mM, and the effect was evaluated by comparing the biomass growth and bioactive compound accumulation. Cultures were maintained in 3 L bubble bioreactors for over 5 weeks. A low ammonium-to-nitrogen ratio was more beneficial for biomass growth; the best were two variants: 5:25 mmol/L and 10:20 mmol/L. These two values were also most beneficial for the accumulation of bioactive compounds. The highest amounts of eleutherosides B and E (291 and 969 µg/L, respectively) were observed when a 10:20 ratio was used (Table 3). For the production of other compounds (chlorogenic acid, total phenolics, and total flavonoids), a 5:25 NH_4_^+^:NO_3_**^−^** ratio was more favorable; the total production of these compounds was 154.30 µg/L [127].

The influence of inoculum size (from 2.5 to 15.0 g/L) on biomass growth and bioactive compound accumulation was also studied for adventitious root cultures in 3 L airlift bioreactors. The highest amount of biomass after a 5-week culture cycle was obtained using 5.0 g/L of inoculum, whereas the smallest inoculum (2.5 g/L) resulted in the fastest growth. Use of inoculum larger than 5.0 g/L resulted in lower biomass weight. Inoculum size also had an impact on the accumulation of bioactive compounds. The highest total content of target compounds (17.53 µg/g DW) was obtained using 5.0 g/L of inoculum (Table 3) [128].

Adventitious roots were treated with various volumes of air during the growth period (0.05–0.4 vvm). The experimental results indicate that an aeration volume of 0.1 vvm is optimal for achieving the highest biomass production and compound accumulation: 60 µg/g DW of eleutheroside B and 108 µg/g DW of eleutheroside E (Table 3). This volume of supplied air allowed us to obtain a high amount of compounds, low rates of root death, and no physiological disorders (root darkening or elongation inhibition) [128].

Temperature is another important parameter for biomass growth and accumulation of bioactive compounds under in vitro conditions. Shohael et al. tested the effect of temperature (12, 16, 24, and 30 °C) on bioreactor cultures of somatic embryos of ES. The optimal temperature for biomass growth was 24 °C. This temperature was also optimal for the accumulation of eleutheroside B (21 µg/g DW), eleutheroside E1 (40 µg/g DW), and chlorogenic acid (Table 3). However, the highest amount of eleutheroside E was accumulated in cultures maintained at a temperature of 12 °C (43 µg/g DW). The highest temperature caused a significant decrease in biomass growth and metabolite accumulation. The authors suggested that the decrease in biomass growth at a temperature of 30 °C is associated with a weakening in protective enzyme activity [122,129].

Another important factor in the cultivation of in vitro cultures is the type of light used. Experiments on ES somatic embryo cultures maintained under fluorescent, blue, red, and blue plus far-red light and in darkness have shown that light conditions do not significantly affect biomass growth. Nevertheless, light conditions influenced the production of active compounds; the accumulation of eleutherosides E and E1 was higher in cultures grown under red light (55 µg/g DW and 50 µg/g DW), and the accumulation of eleutheroside B was higher in the presence of blue light (28 µg/g DW) (Table 3) [122].

Similar studies were carried out on plantlets grown in vitro using 16/8 h illumination with red, blue, and far-red LED lights and a wide-spectrum white fluorescent lamp. The plantlet length was greatest under red and blue light. The leaf area, root length, and fresh weight were highest under blue light. In addition, the accumulation of eleutherosides B and E in the biomass was greatest under blue light. For eleutheroside E1 production, a wide range of white light was the most beneficial [130].

Oxygen (O_2_) and carbon dioxide (CO_2_) are crucial for plant growth, both in vitro and in vivo, and can influence the production of secondary metabolites. Increases in O_2_ or CO_2_ levels often stimulate the production of bioactive compounds. However, the experiments conducted on ES embryogenic suspension cultures maintained in airlift bioreactors showed no significant effect [131].

The influence of plant growth regulators such as gibberellic acid (GA_3_) at different concentrations (0, 1, 2, 3, 4, and 8 mg/L) on embryogenic suspensions of ES cultures was also studied. The use of a concentration of 4.0 mg/L was the most beneficial for both the increase in biomass and the accumulation of eleutheroside B (41 µg/g DW), eleutheroside E (73 µg/g DW), eleutheroside E1 (77 µg/g DW), and chlorogenic acid (Table 3) [131].

Studies have also been conducted to increase the production of bioactive compounds using elicitors in embryogenic suspension cultures of ES. The effect of methyl jasmonate (MJ) at concentrations of 50–400 µmol/L on the production of eleutherosides and chlorogenic acid was tested. The concentration of 200 µmol/L MJ was the most beneficial, causing a 7.3-fold increase in the total content of eleutherosides (eleutheroside B, 37 µg/g DW; eleutheroside E, 99 µg/g DW; and eleutheroside E1, 659 µg/g DW; representing 1.4-, 3.4-, and 14.9-fold increases compared to the control, respectively) and a 3.9-fold increase in chlorogenic acid content (Table 3). At the same time, MJ addition caused a significant decrease in biomass growth [132].

The effects of two elicitors, MJ and salicylic acid (SA), were also tested on adventitious roots in 3 L airlift bioreactors. The growth cycles lasted 6 weeks, and elicitors were added to the medium at concentrations of 50, 100, 200, and 400 µmol/L one week before harvest. The accumulation of eleutherosides B and E, chlorogenic acid, total phenolics, and flavonoids was determined. The highest content of the investigated compounds (except eleutheroside B) was observed after the addition of 50 µmol/L MJ (Table 4). Elicitation using SA had a negative effect on the accumulation of all tested compounds, except eleutheroside B. The accumulation of eleutheroside B increased with increasing SA concentrations up to 2330 µg/L at 400 µmol/L. The highest increase in biomass was observed in cultures without the addition of elicitors. The presence of MJ or SA in the medium had a negative effect on biomass growth, except for the addition of 50 µmol/L MJ, which did not have a significant effect on culture growth. The addition of SA at any concentration inhibited the growth of roots; at a concentration of 400 µmol/L, biomass was over 64% lower than under control conditions. MJ at a concentration of 50 µmol/L was indicated as the optimal elicitor for biomass growth and bioactive compound accumulation. The total production of investigated compounds (in mg/L of medium) was 37.77% higher than under control conditions [125].

**Table 3:** Content of eleutherosides in cultures of somatic embryos.

Culture Conditions		Eleutheroside (µg/L)			References
		B	E	E1	
Bioreactor type	Balloon	327	533	388	[122]
	Bulb	194	550	299	
	Cone	223	418	198	
	Cylinder	164	291	136	
	500 L horizontal drum-type airlift	187	364	231	
	500 L balloon-type airlift	220	413	262	
Aeration volume (vvm)	0.05	205	456	194	
	0.1	257	442	267	
	0.2	210	428	214	
	0.3	190	313	183	
	0.05/0.1/0.2/0.3	290	593	341	
Inoculum density (g/L)	1	139	538	287	
	3	225	526	277	
	5	243	580	377	
	7	229	434	307	
	9	169	242	274	
Temperature (°C)	12	ND	43.1	12.7	[129,131]
	18	15.9	26.9	11.7	
	24	21.2	42.0	39.6	
	30	ND	ND	ND	
Light conditions	Dark	21.3	26.7	39.7	[131]
	Fluorescent	23.1	42.9	48.6	
	Blue	27.9	25.0	24.6	
	Red	14.9	54.5	50.4	
	Blue + far red	22.6	37.2	35.8	
Giberelic acid (mg/L)	0.0	20.1	28.9	40.5	
	1.0	31.2	35.4	55.4	
	2.0	43.1	71.6	73.7	
	3.0	45.2	75.4	74.9	
	4.0	41.0	72.9	77.1	
	8.0	25.5	39.9	17.9	
Methyl jasmonate(µmol/L)	0	25.55	28.60	88.70	[132]
	50	26.73	89.05	235.74	
	100	27.95	93.95	271.90	
	150	32.70	89.00	437.20	
	200	37.40	99.40	649.95	
	300	33.05	90.90	366.45	
	400	31.51	85.26	276.33	

**Table 4:** Content of bioactive compounds in adventitious roots cultures.

Culture Conditions		Bioactive Compounds µg/L						References
		Eleutheroside B	Eleutheroside E	ChlorogenicAcid	Total Phenolics	Total Flavonoids	Total Target Compounds	
Elicited adventitious roots	Contr.	276.07	916.14	66.95	97.88	54.59	220.61	[125]
	MJ 50	275.80	1193.68	78.22	154.91	69.33	303.93	
	MJ 100	258.91	1178.01	75.73	146.77	65.29	289.22	
	MJ 200	249.64	1124.45	62.03	136.90	61.31	261.61	
	MJ 400	211.83	758.91	45.50	120.43	55.76	222.66	
	SA 50	448.32	919.04	65.96	100.24	54.53	222.10	
	SA 100	937.85	878.41	62.24	92.37	50.87	207.30	
	SA 200	1761.62	609.74	38.18	65.21	31.83	137.60	
	SA 400	2329.67	294.15	14.61	44.04	22.39	83.67	
MS medium salt strength	1/4	181.39	460.03	24.68	48.50	25.98	99.80	[126]
	1/2	296.96	757.91	19.00	43.83	21.48	85.36	
	3/4	283.51	825.45	16.25	37.21	16.63	71.19	
	1	222.51	675.57	11.47	31.87	12.33	56.57	
	2	161.43	414.40	11.73	29.38	11.72	53.41	
NH_4_^+^:NO_3_^−^ ratio (mmol/L)	0:30	153.99	488.19	35.86	49.02	29.94	115.46	[127]
	5:25	241.30	830.88	44.31	71.48	37.44	154.30	
	10:20	291.03	969.11	20.89	58.83	31.01	111.00	
	15:15	245.85	897.45	10.72	39.13	15.27	66.26	
	20:10	213.05	729.44	6.65	31.39	9.39	48.25	
	25:5	143.65	368.29	1.21	17.75	4.58	24.05	
	30:0	55.41	147.44	0.44	10.15	1.11	11.90	
Inoculum (g/L)	2.5	46.21	108.53	2.69	9.95	4.60	17.40	[128]
	5.0	27.59	106.61	2.42	10.06	4.91	17.53	
	7.5	26.42	108.89	2.71	9.12	4.03	15.99	
	10.0	15.95	87.47	2.64	9.38	4.16	16.28	
	15.0	6.71	89.65	0.99	8.26	3.41	12.76	
Aeration volume (vvm)	0.4	49.35	106.88	3.02	9.09	5.34	17.60	
	0.05	59.25	107.70	3.36	9.84	5.52	18.88	
	0.1	59.55	108.23	3.34	9.64	5.26	18.41	
	0.2	49.05	90.68	1.44	9.72	5.10	16.39	
	0.05–0.4	52.65	98.78	2.96	9.58	5.20	17.89	

## 8. Conclusions

*Eleutherococcus senticosus*, commonly known as Siberian ginseng, is an adaptogenic plant with a rich history of use in traditional medicine, particularly in Asia. It belongs to the Araliaceae family and is valued for its diverse pharmacological activities. ES is undoubtedly one of the most popular species of useful plants, valued in the pharmaceutical industry as well as in the food and cosmetics industries. The popularity of this species makes it increasingly difficult to obtain in natural locations, which is why modern scientific research is looking for alternative sources of raw material related to the development of research in the field of plant biotechnology focused on this species.

The most important raw materials obtained from ES are rhizomes with roots; however, research is being conducted on the chemical composition and health-promoting properties of the remaining parts of the plant.

Numerous scientific studies have demonstrated the specific chemical composition of this raw material, which is dominated by eleutherosides, glycoproteins, and polysaccharides. ES extract is one of the most commonly used agents because of its adaptogenic and immunostimulatory effects. Several aspects of ES activities have been revealed by the current research data. Research continues to explore its potential health benefits and optimize cultivation methods, underscoring its significance in both traditional and modern medicine.

Although numerous studies have demonstrated ES’s bioactivity, the precise pharmacological mechanisms of its active components have yet to be fully elucidated. To determine these mechanisms, additional pharmacological models capable of depicting and clarifying the actions of ES’s constituents should be developed. Moreover, advanced technologies, such as bioinformatics and various “-omics” approaches (e.g., methods like NMR or HPLC-MS), will be invaluable for building a comprehensive understanding of the complex molecular processes underlying the effects of *E. senticosus*. Finally, further in vitro and in vivo investigations integrating detailed phytochemical profiling, bioavailability and toxicity studies, and metabolomics-driven approaches will be essential to unravel both the individual actions and the interactions of its bioactive compounds.

Undoubtedly, ES will continue to gain more and more attention as a crude and adaptogenic medication. It has been proposed that the numerous active substances isolated from various components of ES have a variety of therapeutic benefits, including anti-inflammatory, anticancer, antidiabetic, neuroprotective, antioxidative, immunomodulatory, anti-leukemic, cardiovascular and cerebrovascular, hypoglycemic, and antiulcer effects. However, the results thus far mainly stem from in vitro or in vivo animal experiments, and the outcomes may not always apply to human contexts. Further clinical trials are required to better evaluate the potential of ES in the prevention and treatment of human diseases and its economic viability.

Additionally, the development of novel ES-based supplements and medications should be promoted if safe and scientific clinical trials are actively carried out. This will allow more people to eventually benefit from their numerous biological activities. It is important to remember that ES raw materials must first be properly purified and standardized before being scaled up for industrial use.

## Data Availability

No new data were created or analyzed in this study. Data sharing is not applicable to this article.

## Supplementary Materials

The following supporting information can be downloaded at: https://www.mdpi.com/article/10.3390/molecules30122512/s1. Table S1: Pharmacological activities of ES [133,134,135,136,137,138,139,140,141,142,143,144,145,146,147,148,149,150,151,152,153,154,155,156,157,158,159,160,161,162,163,164,165,166,167,168,169,170,171,172,173,174,175,176,177,178,179].
