# Supplementary material for: Eleutherococcus senticosus (Acanthopanax senticosus): An Important Adaptogenic Plant
Source: Molecules. 2025 Jun 8;30(12):2512. doi: 10.3390/molecules30122512 (PMC12195798; doi:10.3390/molecules30122512)
Supplement: Supplementary file 1 [file molecules-30-02512-s001.zip › molecules-3649437-supplementary.pdf]

molecules

Supplementary Materials for

Eleutherococcus senticosus (*Acanthopanax senticosus*): An Important Adaptogenic Plant

Grzegorz Kos[1], Katarzyna Czarnek[2,*], Ilona Sadok[3], Agnieszka Krzyszczak-Turczyn[3], Paweł Kubica[1], Karolina Fila[4], Gizem Emre[5], Małgorzata Tatarczak-Michalewska[6], Małgorzata Latalska[7], Eliza Blicharska[6], Daniel Załuski[8], Nazım Şekeroğlu[9,10], Agnieszka Szopa[1,*]

[1] Department of Medicinal Plant and Mushroom Biotechnology, Faculty of Pharmacy, Jagiellonian University, 9 Medyczna St., 30-688 Kraków, Poland; grzegorz.kos@student.uj.edu.pl (G.K.); p.kubica@uj.edu.pl (P.K.)
[2] Institute of Medical Science, Faculty of Medical, The John Paul II Catholic University of Lublin, Konstantynów 1 H St., 20-708 Lublin, Poland
[3] Department of Biomedical and Analytical Chemistry, Institute of Biological Sciences, Faculty of Medicine, Collegium Medicum, The John Paul II Catholic University of Lublin, Konstantynów 1J St., 20-708 Lublin, Poland; ilona.sadok@kul.pl (I.S.); agnieszka.krzyszczak@kul.pl (A.K.-T.)
[4] Institute of Agrophysics, Polish Academy of Sciences, Doświadczalna 4 St., 20-290 Lublin, Poland; k.fila@ipan.lublin.pl (K.F.)
[5] Department of Pharmaceutical Botany, Faculty of Pharmacy, University of Marmara, 34854 İstanbul, Türkiye; gizem.bulut@marmara.edu.tr (G.E.)
[6] Department of Pathobiochemistry and Interdisciplinary Applications of Ion Chromatography, Medical University of Lublin, 1 Chodźki St., 20-093 Lublin, Poland; malgorzata.tatarczak-michalewska@umlub.pl (M.T.-M.); eliza.blicharska@umlub.pl (E.B.)
[7] Chair and Department of General and Pediatric Ophthalmology, Medical University of Lublin, 20-079 Lublin, Poland; malgorzata.latalska@umlub.pl (M.L.)
[8] Department of Pharmaceutical Botany and Pharmacognosy, Ludwik Rydygier Collegium Medicum, Nicolaus Copernicus University, 9 Marie Curie-Skłodowska St., 85-094 Bydgoszcz, Poland; daniel.zaluski@cm.umk.pl (D.Z.)
[9] Department of Biology, Faculty of Science and Literature, Gaziantep University, 27310 Gaziantep, Türkiye; nazimsekeroglu@gantep.edu.tr (N.S.)

* Correspondence: katarzyna.czarnek@kul.pl (K.C.); a.szopa@uj.edu.pl (A.S.)

 https://doi.org/10.3390/molecules30122512

Table S1. Pharmacological activities of ES.

Pharmacological activities	Extracts/Compounds	Administration route	Model	Doses or concentrations tested	Results	Mechanism	References
**Adaptogenic**	Aqueous extract	i.g.	*In vivo*; male C57 BL/6J strain mice	1 g/kg/day	Significant increase in swimming time in mice, inhibition of reduction in NK activity and increase in corticosterone induced by forced swimming	NM	[1]
	ES capsule containing of 0.11% of eleutheroside B (syringin) and 0.12% of eleutheroside E	i.g.	*In vivo*; human	400 mg/ twice a day	Enhanced endurance capacity, elevated cardiovascular functions and altered the metabolism for sparing glycogen in recreationally trained males	NM	[2]
	Aqueous extract	i.g.	*In vivo*; male Kunming mice	100, 200, 400 mg/kg/day	Extension of the swimming time to exhaustion of the mice, increase the tissue glycogen contents, decrease the blood lactate and serum urea nitrogen contents	NM	[3]
	80% Ethanol extract	i.g.	*In vivo*; male Wistar rats	3, 30, 300 mg/kg/ twice a day	Significantly reducing hippocampal CA1 neuronal death by 3.5%, 25.9%, and 53.1%, at 300 mg/kg also decreased ischemia-induced activation and blocked 81.9% of the decrease in spontaneous alternation induced 4-VO	Inhibition of cyclooxygenase-2 (COX-2) expression, microglia and astrocyte expression	[4]
	Aqueous extract	-	*In vitro*; mouse BV-2 microglial	20 μg/mL	Increase of HO-1 expression reduced LPS-induced nitric oxide/ROS production	p38-CREB and Nrf2 pathways	[5]

			cells and mouse HT22 hippocampal cells		in BV2 cells, induction of HO-1 expression protected cells against glutamate-induced neuronal cell death		
**Antidiabetic**	Eleutheroside B	i.v.	*In vivo*; STZ-diabetic rats	1 mg/kg	Syringin reduced plasma glucose levels in a dose-dependent manner. A dose of 1.0 mg/kg significantly attenuated the increase in plasma glucose and stimulated glucose uptake in the soleus muscle of STZ-diabetic ratsdependent on the dose (0.01 to 10.0 micromole/L)	NM	[6]
	50% Ethanol extract	i.g.	*In vivo*; male db/db mice	0.05%, 0.1%	Enhancing glucose uptake, improving insulin resistance and pancreatic islet cell function, regulating glucose metabolism	NM	[7]
	Aqueous extract	i.g.	*In vivo*; male db/db mice	500 mg/kg	ASE inhibited intracellular DG uptake chiefly by inhibiting transport via a glucose transporter	Inhibit α-glucosidase activity in the small intestine	[8]
	60% Ethanol extract	-	*In vitro*	1 mg/mL	Inhibition of the α-glucosidase activity by 73.83%	NM	[9]
	(7S, 8R)-3-hydroxyl-4-meth-oxyl-bal anophonin	-	*In vitro*	IC_{50}: 15.2 ± 1.4 μM	Discovery of new PTP1B inhibitors and providing the basis for the treatment of type-2 diabetesas well as obesity	NM	[10]
**Neuroprotective**	Aqueous extract	i.g.	*In vivo*; mice	2000 mg/kg	Significantly reduction of the duration of immobility in both the forced swimming test and the tail suspension test, significantly increase of the levels of 5-HT,	Depressive mechanism mediated by the central	[11]

					NE, and DA in the whole brain of mice, significantly up-regulated the level of CREB protein, antidepressant-like properties	monoaminergic neurotransmitter system and CREB protein expression	
	Aqueous extract	i.g.	*In vivo*; SD rats	1%, 5%	Significantly decreased latency to eat, increased the time spent on the open arm, 5% treatment showed a significant decrease in LFnu, significant increase in HFnu, and increase hippocampal BDNF protein expression	Autonomic regulation function and activate hippocampal BDNF–TrkB signaling	[12]
	Aqueous extract	i.g.	*In vivo*; mice	235.7 mg/kg/day	ES extract had positive effects on nerve cells' structure, adhesion, locomotion, fission, and phagocytosis, through regulating various action pathways to maintain normal mouse neurological activity	Hippo, phagosome, PI3K/AktNeurotrophin, Rap1, gap junction glycolysis/gluconeogenesis, and HIF-1 signaling pathways	[13]
	Aqueous extract	i.p.	*In vivo*; SD rats	100 mg/kg	Reduction of the infarct volume by 36.6% compared with the control and down-regulation of COX-2 and OX-42 expression in the penumbral region at 24 h after MCAo	Inhibition of inflammation and microglia activation in brain ischaemia	[14]
	80% Ethanol extract	i.g.	*In vivo*; C57BL/6 mice	5.01, 20.04 mg/kg	Pole-climbing time in low and high dose group were significantly less than model	NM	[15]

					group, increase in striatal dopamine levels, modulation of dopamine D1/D2 receptor expression, inhibition of dopaminergic neuron apoptosis		
	100% Ethanol, 50% ethanol and water	i.g.	*In vivo*; rats	250 mg/kg	Prophylactic effects on MPTP-induced Parkinsonian bradykinesia and catalepsy, ASH provided cytoprotective effects against MPTP-induced loss of dopamine cells	NM	[16]
	100% Ethanol, 50% ethanol and hot water	i.g.	*In vivo*; male SD rats	50, 100, 500 mg/kg	Action on the frontal cortex and anterior hypothalamus of rats to enhance the NA level and its turnover, the long-term administration stimulates DA and its turnover in the striatum and anterior hypothalamus	NM	[17]
	Aqueous extract	i.g.	*In vivo*; rats	20, 50 mg/kg	Saponin library-guided pseudotargeted strategy was established to support the rapid monitoring of 26 blood-brain barrier (BBB)-permeated saponins from ESL-SAP-administered rats, key BBB-transferred saponins primarily interacted with targets HR, MAPK1, and MAPK8	AGE-RAGE signaling pathway and PI3K-Akt signaling pathway	[18]
	Phenylpropanoids	i.g.	*In vivo*; SAMP8 mice	300, 600 mg/kg	Reduce the degenerative characteristics of Alzheimer's disease and enhance cognitive function in SAMP8 mice	Modulating the Mst1/Nrf2/Sirt3 signaling pathway	[19]
	Phenylpropanoi	-	*In vitro*;	NM	Reduction of intracellular ROS levels	Modulating the	

	ds		L-glutamate (L-Glu)-induced HT22 cells			Mst1/Nrf2/Sirt3 signaling pathway	
	Eleutheroside B	-	*In vitro*; cortical neurons of rats	10-1,000 ng/mL	Increase reconstruction of synapses of axons and dendrites	NM	[20]
	Methanol extract Compounds: Eleutheroside B, Eleutheroside E and Isofraxidin	-	*In vitro*; rat cortical neurons	1, 10 μM	The ethyl acetate, n-butanol, and water fractions protected against axonal and dendritic atrophy caused by amyloid beta (Aβ25–35); eleutheroside B, eleutheroside E, and isofraxidin showed the most potent protective effects on axonal and dendritic length	NM	[21]
	Aqueous extract	-	*In vitro*; PC12 cells	25-1600 μg/mL	Significantly improved cell viability, reduced LDH release, inhibited apoptosis, alleviated intracellular Ca^{2+} overload, and increased BDNF mRNA levels and CREB protein expression compared to the corticosterone-treated group	Inhibit LDH release and PC12 cells apoptosis, intracellular Ca^{2+} overloading	[22]
**Anticancer**	Aqueous extract	i.v.	*In vivo*; colon26-M3.1 tumor cells and B16-BL6 melanoma cells in BALB/c and C57BL/6 mice	GF-AS (0.5-50 mg/20 g) or EN-SP (0.2-5 mg/20 g)	Glycoproteins EN-SP fractionated from GF-AS dramatically inhibited metastasis of colon26-M3.1 carcinoma cells to the lung in a dose-dependent manner	Activation of NK cells and macrophages	[23]
	Aqueous extract	-	*In vitro*;MCF-7 and MDA-MB-231 human breast cancer cell lines	50, 100, 250, 500 μg/mL	ES fruit dose-dependently inhibited growth of MDA-MB-231 and MCF-7 cells, downregulated Bcl-xL (and survivin in MCF-7), and upregulated p21, RIP-1 and	Apoptotic cell death	[24]

				other proapoptotic genes		
Aqueous extract	-	*In vitro*;A549 non–small-cell lung cancer cells	0, 100, 150, and 200 mg/mL	ES significantly increased apoptosis in A549 cells with increasing concentration, the mRNA and protein expression of EGFR, MAPK3, and ICAM1 significantly increased, while CTSK decreased	PI3K/Akt, MAPK, NF-κB/TNF pathways	[25]
Polysaccharides	i.p.	*In vivo*; female C57/ BLmice	0.12 mL/piece	Intervene of lung cancer	Decrease of PAI-1	[26]
Polysaccharides	s.c.	*In vivo*; mice, HEPA cells	0.34 g/kg/day	Prolonged life expectancy of mice	NM	[27]
Polysaccharides	s.c.	*In vivo*; mice S37, S180 cells	0.34 g/kg/day	Excited reticuloendothelial system	Immunomodu-lation	
Polysaccharides	i.g.	*In vivo*; Kunming mice	50, 100, 200 mg/kg/day	Increased survival days, regulation of cytokines	Immunomodu-lation	[28]
Polysaccharides	i.g.	*In vivo*; Kunming mice	50, 100, 200 mg/kg/day	Increased cell growth	Apoptosis	[29]
Polysaccharides	i.p.	*In vivo*; C57BL/6 mice	10 mg/kg/day	Increased LAK activity, increased spleno-cyte	NM	[30]
Polysaccharides	i.g.	*In vivo*; male Wistar rats	20%	Inhibit tumor	NM	[31]
Dealcoholized tincture of ES-root mixed with drinking water	i.g.	*In vivo*; female rats	10 mL/L	Prevention of development of both benign and malignant tumors and increased survival of female rats exposed to a single total dose of γ-irradiation of 4 Gy	NM	[32]

	Eleutheroside B	-	*In vitro*; non-tumorigenic (M10), tumorigenic (MCF7) and metastatic (MDA-MB-231) breast cancer cell lines	10, 30 μM	Eleutheroside B caused the up-regulation of p21, cleaved caspase-3/caspases-9 and PARP but downregulated CDK4 and XIAP expression concomitant with the suppression of growth in breast carcinoma cells; induced excessive ROS levels in breast cancer cells	Induce oxidative stress	[33]
	Polysaccharides	-	*In vitro*; human non-small cell lung cancer cell line NCI-H520	10, 20, 40, 80, 160, 320 mg/mL	Inhibited the proliferation, metastasis	Wnt/β-catenin pathways	[34]
	n-Butanol extract	-	*In vitro*; human stomach cancer KATO III cells	23, 45, 90 μM	Growth inhibition and induction of apoptosis (by the formation of apoptotic bodies), DNA fragmentation, sesamin showedthe strongest effect among the tested compounds—chlorogenic acid, syringaresinol di-O-β-D-glucoside, syringin, and sesamin, suppressing cell growth and inducing apoptosis	Apoptosis	[35]
	Sesamin	-	*In vitro*; human breast cancer MCF-7 cell	1, 10, 50 μM	Inhibition of cell proliferation by inducing cell cycle arrest and apoptosis, induction of cell cycle arrest is through the increasing of p53 and Chk2 and apoptosis is through the activation of the Bax and caspase-3 pathways	Modulating apoptotic signal pathways, inhibiting tumor cell growth	[36]
	Isofraxidin, eleutheroside B,	-	*In vitro*; human synovial sarcoma	Isofraxidin (135, 450 μM),	Isofraxidin had significant inhibitory effects on cell growth, SR suppressed the	Suppressing various gene	[37]

	(+)-syringaresinol-di-O-β-D-glucoside (SR)		cell line, SW982	eleutheroside B (17, 50, 150 μM), (+)-syringaresinol-di-O-β-D-glucoside (SR) (17, 50, 150 μM)	production of interleukin-6 (IL-6) at low concentrations, SR and syringin significantly suppressed the production of prostaglandin E2, SR was more potent than syringin and isofraxidin at inhibiting the expression of IL-1b, IL-6, cyclo-oxygenase (COX)-2 and matrix metalloproteinases (MMP)-1 mRNA, syringin at inhibiting the expression of MMP-2, SR significantly reduced MMP-1 promoter luciferase activity and DNA-binding activity of transcriptional factors AP-1 and NF-κB	expression through inhibiting AP-1 and/or NF-κB activities	
	Isofraxidin, eleutheroside E	-	*In vitro*; human hepatoma cell lines HuH-7, HepG2 and HLE, human lung fibroblast cell line IMR90	33, 66, 100 μM	Isofraxidin significantly inhibited hepatoma cell invasion, without affecting cell attachment or growth, isofraxidin showed an inhibitory effect on the phosphorylation of extracellular signal-regulated kinase 1/2 (ERK1/2), inhibited expression of MMP-7 and in vitro cell invasion at a non-toxic level	NM	[38]
	Polysaccharides	-	*In vitro*; H446 cells	240, 480, 960 μg/mL	Decrease cell proliferation	Increase P53, Bax, decrease Bcl-2, p-38 expression EPK pathway, apoptosis	[39]
	Polysaccharides	-	*In vitro*; H446 cells	240, 480, 960	Decrease G2/M arrest	EPK MAP	[40]

				μg/mL		kinase pathways	
	Polysaccharide	-	*In vitro*; A-549 cells	35.7 μM	Decreased cell viability	NM	[41]
	Polysaccharides	-	*In vitro*; HepG2 cells	10, 20, 40, 80 mg/L	Increase apoptosis, G0/G1 phase arrest in	Wnt/β-catenin pathway	[42]
	Polysaccharides	-	*In vitro*; Hela cells	1, 2, 4, 8, 16 mg/mL	Decrease cell proliferation	Increase Bax expression, apoptosis	[43]
	Polysaccharides	-	*In vitro*; Hela cells	1, 2, 4, 8, 16 mg/mL	Decrease cell proliferation	Decreased survivin expression, apoptosis	[44]
	Polysaccharides	-	*In vitro*; S180 cells	1, 10, 100, 500, 1000 mg/L	Decrease cell proliferation	Apoptosis	[45]
	Polysaccharides	-	*In vitro*; Hep 2 CD133	100 mg/L	Decrease CD133 stem cells	Apoptosis	[46]
**Antioxidative**	Aqueous extract	i.g.	*In vivo*; male Wistar rats	50, 100, 200 mg/kg	Decrease of FGB, TBARS and LP levels, increase of the expression level of FINS, SOD and CAT in plasma, liver, kidney and heart, alleviation of oxidative stress and free radicals, and enhancing enzymatic defenses	NM	[47]
	Flavonoids	i.g.	*In vivo*; SPF male C57BL/6 mice	50, 100, 200 g/L	Inhibition of colitis by improving antioxidant activity, maintaining the normal physiological function of the intestinal tract	NM	[48]

Aqueous extract	i.g.	*In vivo*; male SD rats	200 mg/kg/day	Significant increases in antioxidant enzyme activities suchEShepatic cytosolic superoxide dismutase, catalase and glutathione peroxidase by 30.31, 19.82 and 155%, n-butanol fraction showed a 65.79% inhibition of serum GPT activity elevated with hepatic damage induced by CCI4-intoxication, eleutheroside B showed moderate free radical scavenging effect on DPPH	NM	[49]
Aqueous extract compounds: caffeic acid and chlorogenic acid	-	*In vitro*	1 mg/mL	Fruit extracts fromESshowed highest ABTS, FRAP, reducing power and ORAC capacity, leaf extract ofES exhibited strongest DPPH radical scavenging activity	NM	[50]
Aqueous extract	-	*In vitro*; PC12 cells	20, 40, 80, 120, 160, 200 μg/mL	52 different metabolites in the different parts of ESwere significantly related to the H_2O_2-induced oxidative -stress, the roots of ESwas slightly stronger than that of the seeds and leaves in antioxidative activity	NM	[51]
Syringin, eleutheroside E, polysaccharides and flavones	-	*In vitro*	5 mg/mL	Polysaccharides and flavones were more effective in DPPH and ABTS radical scavenging activities than syringin and eleutheroside E	Improve the radical scavenging activities	[52]
Flavonoids	-	*In vitro*; H_2O_2-induced RAW 264.7 cells	15-120 mg/L	Protection of macrophages under oxidative stress by increasing the activity of antioxidant enzymes in the cell and activating the Nrf2/Keap1/HO-1 signaling	NM	[48]

					pathway		
	Polysaccharides	-	*In vitro*; ·OH, O^{2-}	3 mg/L	Eliminate ·OH and O^{2-}	Oxidative stress	[53]
	Polysaccharides	i.g.	*In vivo*; Wistar male rats	50, 100, 200 mg/kg	Decrease oxidative damage	Oxidative stress	[54]
	Polysaccharides	-	*In vitro*; SD rats hippocampal neurons	2.5, 5, 10 μg/mL	Resist oxidative stress damage	Oxidative stress	[55]
	Polysaccharide	-	*In vitro*; Mice spleen cells	0.2 mg/mL	Eliminate ·OH, O2-, DPPH·	Oxidative stress	[56]
	Polysaccharides	-	*In vitro*; SD rats hippocampal neurons	2.5, 5, 10 μg/mL	Reduction of gene expression	NF-κB pathways	[57]
	Polysaccharides	-	*In vitro*; SD rats hippocampal neurons	1.25, 2.5, 5, 10 g/mL	Reduction in the expression of iNOS mRNA	iNOS mRNA expression	[58]
	Aqueous extract compounds: phenolic and flavonoids	-	*In vitro*; RAW 264.7 cell line	IC50:18.21 μg/mL (CH_2Cl_2 fraction) IC50: 8.86 μg/mL (EtoAC fraction) IC50: 47.88 μg/mL (n-BuOH fraction) IC50: 59.74 μg/mL (water fraction)	The CH_2Cl_2 fraction showed the greatest potential for protein protection and significant ability to inhibit nitric oxide production, this fraction also significantly reduced reactive oxygen species levels and effectively suppressed the expression of key inflammatory markers like iNOS, COX-2, tumor necrosis factor-α (TNF-α), and IL-1β, the EtOAC fraction stood out for its strong antioxidant activity	NM	[59]

	Polysaccharides	i.g.	*In vivo*; BALB/c mice	36.25, 72.5,145 mg/kg	Reduction of MDA, NO, IL-1β, and TNF-α activity, increase of GSH-PX and SOD content, decrease of TNF-α, ICAM-1, iNOS, and NF-κB protein expression levels in liver tissues, alleviation of inflammatory cell infiltration, hepatocyte cytoplasm was loose and swollen, and improvement of nuclear condensation and staining	Regulating secretion of inflammatory cytokines and the expression of adhesion factor through NF-κB signaling pathway	[60]
**Immunomodulatory**	Isofraxidin	i.p.	*In vivo*; mice	1, 5, 15 mg/kg	Reduction in the mortality rate, body weight loss, organ coefficient, and histological alterations, inhibition of NF-κB protein expression, serum NO and IL-6 levels, and liver TNF-α production	Regulation of NF-κB signal and down-regulation of production of inflammatory cytokine TNF-α	[61]
	Eleutheroside E	i.g.	*In vivo*; mice	30 mg/kg/day	significantly decreased the inflammatory cell infiltration, pannus formation, cartilage damage, and bone erosion, causing a marked decrease in the production of TNF-α and IL-6	Inhibit cytokine release	[62]
	Polysaccharides	i.p.	*In vivo*; mice	30 mg/kg/day	Prolonged sleep time, increased number of thymocytes	Immunomodulation	[27]
	Polysaccharides	i.p.	*In vivo*; mice	30 mg/kg/day	Prolonged sleep time, increased number of white blood cells	Immunomodulation	
	Polysaccharides	s.c.	*In vivo*; mice	0.067 g/kg/day	Increased formation of antibodies in the	Immunomodul	

				spleen	ation	
Polysaccharides	i.p.	*In vivo*; male BALB/c, C57BL/6 mice	100 mg/kg/day	Stimulate T-cell proliferation	NM	[63]
Polysaccharides	i.p.	*In vivo*; male BALB/c, C57BL/7 mice	100 mg/kg/day	Increased number of antibody-secreting cells, increasedC57BL/7 mice delayed-type hypersensitivity	Immunomodulation	[64]
Polysaccharides	i.p.	*In vivo*; LACA mice	12.5,25,50,100 mg/kg	Increased humoral immune response	NM	[65]
Polysaccharides	i.g.	*In vivo*; Kunming mice	25,50,100 mg/kg	Increased proliferation of splenic lymphocytes, increased CD3+ CD4+/ CD3+ CD8+	Immunomodulation	[66]
Polysaccharides	i.g.	*In vivo*; Kunming mice	25,50,100 mg/kg	Increased he body's cellular immune function	Immunomodulation	[67]
Polysaccharides	i.g.	*In vivo*; BALB/c mice	36,25,72.5,145 mg/kg	Increased phagocytic function of immunosuppressed Mice, increased production of hemolysin HC50	Immunomodulation	[68]
Polysaccharides	i.g.	*In vivo*; ICR mice	6, 12, 24 mg/10g/day	Regulation of serum hemolysin level, and decreased cell apoptosisto enhance the body's humoral immune function	Apoptosis	[69]
Polysaccharides	i.m.	*In vivo*; chickens	100, 200 mg/mL/day	Increasing BW, lymphocyte proliferation, antibody titers, the proportion of CD4+ and CD8+ T cells, and concentration of interferon-gamma and IL-2 in Cy-induced immunosuppressive chickens	NM	[70]
Aqueous extract	i.p.	*In vivo*; female	100, 200, 400	Protection of mice against	NM	[71]

			BALB/c mice	mg/kg	LPS/D-GalN-induced endotoxic shock involving inhibition of NF-κB activation, down-regulation of TNF-α and up-regulation of IL-10		
	Aqueous extract	-	*In vitro*; human peripheral blood mononuclear cells	400 μg/mL	Dose-dependent inhibition of PHA-stimulated production of IL-2, IFN-γ and TNF-α	NM	[72]
	Polysaccharides	-	*In vitro*; B cells, T cells, and macrophages	1-30 μg/mL	Activation of B cells and macrophages by interacting with TLRs and subsequent activation of mitogen-activated protein kinases and NF-κB	NM	[73]
	Extract	-	*In vitro*; human peripheral blood lymphocytes (PBL)	280, 700, 1400 μg/mL	Stimulation of the INF-γ, neopterin, and β2MG formation, increases the formation of TNF-α and β2MG in cultivated whole blood cells	NM	[74]
	Ethanolic liquid extract	-	*In vitro*; human mononuclear cells	0.03-3 mg/mL	Fluid extracts can induce production of IL-1 and IL-6 but not IL-2	NM	[75]
	Polysaccharides	-	*In vitro*; weaned piglets	800 mg/kg	Modulate the release of pro-inflammatory cytokines	Cytokine expression	[76]
	Polysaccharides		*In vitro*; RAW 264.7 cells	50, 100, 200, 400, 600, and 800 μg/mL	Cytokine up-regulation (increases iNOS, IL-1β, IL-6 and TNF-α levels in activated macrophages),TLR4 mediates iNOS, TNF-α and IL-6 expression, TLR2 mediates TNF-α and IL-1β expression	Activating the TLR/MAPK/ NF-κB signaling pathway	[77]
**Anti-leukemic**	Polysaccharides	i.p.	*In vivo*; mice, L615 cells	0.34 g/kg/day	No significant effect on survival time	Oxidative stress	[27]
	Polysaccharides	-	*In vitro*; K562 cells	1, 10, 100, 500,	Decrease cell proliferation	Apoptosis	[45]

				1000 mg/L			
	Polysaccharides	-	*In vitro*; K562 cells	0.405, 0.810, 1.620, 2.430, 3.240 mg/mL	Increase K562 cell apoptosis	Apoptosis	[78]
	Polysaccharide	-	*In vitro*; P-388 cells	35.69 μM	Decrease mouse white blood cell activity	-	[41]
**Anti-inflammatory**	Total flavonoids	i.v.	*In vivo*; SD rats	3.5 mg/100 g	No effect on mortality reduction, ES and 3-methyladenine had a borderline mortality rate in rats, changed pancreatic damage, high doses of lipopolysaccharide can cause pancreatic tissue damage	Inhibiting the PI3K/Akt or NF-κB signaling pathway, inhibiting abnormal autophagy activation of pancreatic acinar cells	[79]
	Total flavonoids (60% ethanol extract)	i.g.	*In vivo*; mice	200, 400, 800 mg/kg	Inhibiting intestine injury, mitigating inflammation, preserving the integrity of the intestinal barrier and regulating gut microbiota homeostasis	TLR4/NF-κB signaling pathway	[80]
	Polysaccharides	i.g,	*In vivo*; Kunming mice	20 mg/kg/day	Improve immune cytokines	Cytokine expression	[28]
	AS	i.v.	*In vivo*; C57/BL6 male mice	20 mg/kg	Reduction of the levels of tumor necrosis factor (TNF)-α, interleukin (IL)-6, and neutrophils in bronchoalveolar lavage fluid, weakening of inflammation in lung tissues, decreased the level of NF-κB and the DNA-binding activity of NF-κB	NF-κB/MLCK pathway	[81]

	Polysaccharides	i.g.	*In vivo*; BALB/c mice	36.25,72.5,145 mg/kg	Decreased the number of enzymes, protects liver	Antioxidant index	[82]
	Polysaccharides	i.g.	*In vivo*; Wistar male rats	50,100,200 mg/kg	Decreased inflammatory cytokines	NM	[54]
	Polysaccharides	i.g.	*In vivo*; male BALB/c mice	14.5 mg/mL/day	Improve the body's immunity, anti-inflammatory, antioxidant, and supply the body's energy	Glutathione metabolism, purine generation metabolism, cysteine, methionine metabolism pathways	[83]
	Polysaccharides	i.g.	*In vivo*; male and female Drosophila flies	15, 30 mg/mL	Reduction of the enhanced deaths of intestinal epithelial cells and the excessive release of ROS and AMP induced by SDS, increase the survival rate of Drosophila adults under SDS-feeding condition, ESPS can regulate DSS-induced excessive ISC proliferation and differentiation, ESPS can prolong the lifespan of female flies but not male flies	Multiple signaling EGFR, JNK, and Notch pathways	[84]
	Polysaccharides	i.g.	*In vivo*; BALB/C mice	300 mg/kg/day	Pretreatment with oral ESPS prior to the development of endotoxemia can mitigate intestinal epithelial TJ breakdown in the mouse model of endotoxemia	Inhibition of activation of the NF-κB/MLCK signaling pathway	[85]

Polysaccharides	i.g.	*In vivo*; male BALB/c mice	2.0, 4.6, 6.9 mg/mL	Decreased liver cell damage	NM	[83]
Polysaccharides	i.g.	*In vivo*; BALB/c mice	36.25,72.5,145 mg/kg	Decreased secretion and expression of inflammatory cytokines	Cytokine expression	[82]
Polysaccharides	i.g.	*In vivo*; BALB/c mice	36.25,72.5,145 mg/kg	The activity of inflammatory cytokines, decreased adhesion factors, decreased secretion and expression of inflammatory cytokines	Cytokine expression	[60]
Polysaccharides	i.g.	*In vivo*; KM mice	14.5 mg/mL/day	Enhancing the body's immune function, scavenging free radicals, decreased liver cell apoptosis	ES attenuate NF-κB–driven inflammation (reducing pro-inflammatory cytokines and adhesion molecules, while boosting IL-10)	[86]
Total flavones	-	*In vitro*; rat ventricular myocytes	300 μg/mL	Significant inhibition of the Ca^{2+} transient, decrease the contractility of cardiomyocytes, leading to the restraint of Ca^{2+} flow into cardiomyocytes and decrease in $[Ca^{2+}]i$	Inhibition of L-type Ca^{2+} channel	[87]
Aqueous extract compounds: phenolic and flavonoids	-	*In vitro*; RAW 264.7 cell line	IC_{50}:18.21 μg/mL (CH_2Cl_2 fraction) IC_{50}: 8.86 μg/mL	The CH_2Cl_2 fraction showed the greatest potential for protein protection and significant ability to inhibit nitric oxide production, this fraction also significantly	NM	[59]

				(EtoAC fraction) IC_{50}: 47.88 μg/mL (n-BuOH fraction) IC_{50}: 59.74 μg/mL (water fraction)	reduced reactive oxygen species levels and effectively suppressed the expression of key inflammatory markers like iNOS, COX-2, TNF-α, and IL-1β, the EtOAC fraction stood out for its strong antioxidant activity		
**Cardiovascular and cerebrovascular systems**	60% Ethanol extract	i.g.	*In vivo*; SD rats	100 mg/kg	Three predicted target proteins, such-COX-2, monoamine oxidase (MAO), and nitric oxide synthase (NOS), were selectedthe potential target enzymes for ES in ischemic stroke treatment	NM	[88]
	Saponins	i.v.	*In vivo*; rats	25, 50, 100 mg/kg	Improving free radicals and myocardial metabolism, decreasing plasma ET, Ang II and TXA2 levels, increasing plasma PGI2 level and PGI2/TXA2 ratio, serum LPO content declined, significantly increasing SOD and GSH-Px activity	NM	[89]
	Eleutheroside B	-	*In vitro*; neonatal rat cardiomyocytes	400 μg/mL	AS-B demonstrates protective effects against oxidative damage by reducing lipid peroxidation and enhancing antioxidant defense mechanisms	NM	[90]
	Aqueous extract	-	*In vitro*; mouse brain cells	235.7 mg/kg/day	Potential to mitigate radiation-induced brain injury by reducing oxidative stress, enhancing neuroprotection, regulating protein expression and signaling pathways to maintain brain function	NM	[91]

**Antiulcer**	Aqueous extract	i.g.	*In vivo*; male Wistar rats	50, 100, 500 mg/kg	Pre-administration in a dose of 500 mg/kg showed the most potent inhibition without affecting either body or adrenal glands weights, chlorogenic acid and syringaresinol di-o-β-D-glucoside showed a significantly inhibitory effect on gastric ulcer (21.4% and 51.3%, respectively)	NM	[92]

i.g. - intragastric administration; i.p. - intraperitoneal injection; i.v. - intravenous injection; s.c. - subcutaneous injection; i.m. - intramuscular injection; SD rats - Sprague-Dawley rats; ESPS - *Eleutherococcussenticosus* polysaccharides; NM - not mentioned; STZ-diabetic rats - streptozotocin-induced diabetic rats; IC_{50} - half maximal inhibitory concentration

References

1. Kimura, Y.; Sumiyoshi, M. Effects of Various Eleutherococcus Senticosus Cortex on Swimming Time, Natural Killer Activity and Corticosterone Level in Forced Swimming Stressed Mice. *J Ethnopharmacol* **2004**, *95*, 447–453, doi:10.1016/j.jep.2004.08.027.
2. Kuo, J.; Chen, K.W.C.; Cheng, I.S.; Tsai, P.H.; Lu, Y.J.; Lee, N.Y. The Effect of Eight Weeks of Supplementation with Eleutherococcus Senticosus on Endurance Capacity and Metabolism in Human. *Chinese Journal of Physiology* **2010**, *53*, 105–111, doi:10.4077/CJP.2010.AMK018.
3. Zhang, X.L.; Ren, F.; Huang, W.; Ding, R.T.; Zhou, Q.S.; Liu, X.W. Anti-Fatigue Activity of Extracts of Stem Bark from Acanthopanax Senticosus. *Molecules* **2011**, *16*, 28–37, doi:10.3390/molecules16010028.
4. Lee, D.; Park, J.; Yoon, J.; Kim, M.Y.; Choi, H.Y.; Kim, H. Neuroprotective Effects of Eleutherococcus Senticosus Bark on Transient Global Cerebral Ischemia in Rats. *J Ethnopharmacol* **2012**, *139*, 6–11, doi:10.1016/j.jep.2011.05.024.
5. Jin, M.L.; Park, S.Y.; Kim, Y.H.; Park, G.; Lee, S.J. Acanthopanax Senticosus Exerts Neuroprotective Effects through HO-1 Signaling in Hippocampal and Microglial Cells. *Environ Toxicol Pharmacol* **2013**, *35*, 335–346, doi:10.1016/j.etap.2013.01.004.
6. Niu, H.-S.; Liu, I.-M.; Cheng, J.-T.; Lin, C.-L.; Hsu, F.-L. Hypoglycemic Effect of Syringin from Eleutherococcus Senticosus in Streptozotocin-Induced Diabetic Rats. *Planta Med* **2008**, *74*, 109–113.
7. Ahn, J.; Um, M.Y.; Lee, H.; Jung, C.H.; Heo, S.H.; Ha, T.Y. Eleutheroside E, an Active Component of Eleutherococcus Senticosus, Ameliorates Insulin Resistance in Type 2 Diabetic Db/Db Mice. *Evidence-based Complementary and Alternative Medicine* **2013**, *2013*, doi:10.1155/2013/934183.
8. Watanabe, K.; Kamata, K.; Sato, J.; Takahashi, T. Fundamental Studies on the Inhibitory Action of Acanthopanax Senticosus Harms on Glucose Absorption. *J Ethnopharmacol* **2010**, *132*, 193–199, doi:10.1016/J.JEP.2010.08.014.
9. Zhou, H.; Xing, J.; Liu, S.; Song, F.; Cai, Z.; Pi, Z.; Liu, Z.; Liu, S. Screening and Determination for Potential α-Glucosidase Inhibitors from Leaves of Acanthopanax Senticosus Harms by Using UF-LC/MS and ESI-MS n. *Phytochemical Analysis* **2012**, *23*, 315–323, doi:10.1002/pca.1360.
10. Li, J.L.; Li, N.; Xing, S.S.; Zhang, N.; Li, B.B.; Chen, J.G.; Ahn, J.S.; Cui, L. New Neo-Lignan from Acanthopanax Senticosus with Protein Tyrosine Phosphatase 1B Inhibitory Activity. *Arch Pharm Res* **2017**, *40*, 1265–1270, doi:10.1007/s12272-015-0659-7.
11. Jin, L.; Wu, F.; Li, X.; Li, H.; Du, C.; Jiang, Q.; You, J.; Li, S.; Xu, Y. Anti-Depressant Effects of Aqueous Extract from Acanthopanax Senticosus in Mice. *Phytotherapy Research* **2013**, *27*, 1829–1833, doi:10.1002/ptr.4938.
12. Miyazaki, S.; Oikawa, H.; Takekoshi, H.; Hoshizaki, M.; Ogata, M.; Fujikawa, T. Anxiolytic Effects of Acanthopanax Senticosus HARMS Occur via Regulation of Autonomic Function and Activate Hippocampal BDNF-TrkB Signaling. *Molecules* **2019**, *24*, 1–15, doi:10.3390/molecules24010132.
13. Zhou, A.Y.; Song, B.W.; Fu, C.Y.; Baranenko, D.D.; Wang, E.J.; Li, F.Y.; Lu, G.W. Acanthopanax Senticosus Reduces Brain Injury in Mice Exposed to Low Linear Energy Transfer Radiation. *Biomedicine and Pharmacotherapy* **2018**, *99*, 781–790, doi:10.1016/j.biopha.2018.01.001.
14. Bu, Y.; Jin, Z.H.; Park, S.Y.; Baek, S.; Rho, S.; Ha, N.; Park, S.K.; Kim, S.Y.; Kim, H. Siberian Ginseng Reduces Infarct Volume in Transient Focal Cerebral Ischaemia in Sprague-Dawley Rats. *Phytotherapy Research* **2005**, *19*, 167–169, doi:10.1002/ptr.1649.
15. Liu, S.M.; Li, X.Z.; Huo, Y.; Lu, F. Protective Effect of Extract of Acanthopanax Senticosus Harms on Dopaminergic Neurons in Parkinson's Disease Mice. *Phytomedicine* **2012**, *19*, 631–638, doi:10.1016/j.phymed.2012.02.006.

16. Fujikawa, T.; Miguchi, S.; Kanada, N.; Nakai, N.; Ogata, M.; Suzuki, I.; Nakashima, K. Acanthopanax Senticosus Harms as a Prophylactic for MPTP-Induced Parkinson's Disease in Rats. *J Ethnopharmacol* **2005**, *97*, 375–381, doi:10.1016/j.jep.2004.11.031.
17. Fujikawa, T.; Soya, H.; Hibasami, H.; Kawashima, H.; Takeda, H.; Nishibe, S.; Nakashima, K. Effect of Acanthopanax Senticosus Harms on Biogenic Monoamine Levels in the Rat Brain. *Phytotherapy Research* **2002**, *16*, 474–478, doi:10.1002/ptr.1024.
18. Huang, Y.-H.; Ding, W.-L.; Li, X.-T.; Cai, M.-T.; Li, H.-L.; Yang, Z.-Y.; Piao, X.-H.; Zhu, S.; Tohda, C.; Komatsu, K.; et al. Memory Enhancement Effect of Saponins from Eleutherococcus Senticosus Leaves and Blood–Brain Barrier-Permeated Saponins Profiling Using a Pseudotargeted Monitoring Strategy. *Food Funct* **2022**, *13*, 3603–3620.
19. Yang, R.; Meng, X.; Zhao, W.; Xu, S.Q.; Wang, S.Y.; Li, M.M.; Guan, W.; Chen, Q.S.; Zhang, L.L.; Kuang, H.X.; et al. Phenylpropanoids of Eleutherococcus Senticosus (Rupr. & Maxim.) Maxim. Alleviate Oxidative Stress in Alzheimer's Disease in Vitro and in Vivo Models by Regulating Mst1 and Affecting the Nrf2/Sirt3 Pathway. *Bioorg Chem* **2025**, *159*, 108347, doi:10.1016/J.BIOORG.2025.108347.
20. Tohda, C.; Ichimura, M.; Bai, Y.; Tanaka, K.; Zhu, S.; Komatsu, K. Inhibitory Effects of Eleutherococcus Senticosus Extracts on Amyloid β(25-35)-Induced Neuritic Atrophy and Synaptic Loss. *J Pharmacol Sci* **2008**, *107*, 329–339, doi:10.1254/jphs.08046FP.
21. Bai, Y.; Tohda, C.; Zhu, S.; Hattori, M.; Komatsu, K. Active Components from Siberian Ginseng (Eleutherococcus Senticosus) for Protection of Amyloid β(25-35)-Induced Neuritic Atrophy in Cultured Rat Cortical Neurons. *J Nat Med* **2011**, *65*, 417–423, doi:10.1007/s11418-011-0509-y.
22. Wu, F.; Li, H.; Zhao, L.; Li, X.; You, J.; Jiang, Q.; Li, S.; Jin, L.; Xu, Y. Protective Effects of Aqueous Extract from Acanthopanax Senticosus against Corticosterone-Induced Neurotoxicity in PC12 Cells. *J Ethnopharmacol* **2013**, *148*, 861–868, doi:10.1016/j.jep.2013.05.026.
23. Ha, E.S.; Hwang, S.H.; Shin, K.-S.; Yu, K.-W.; Lee, K.-H.; Choi, J.S.; Park, W.-M.; Yoon, T.J. Anti-Metastatic Activity of Glycoprotein Fractionated from Acanthopanax Senticosus, Involvement of NK-Cell and Macrophage Activation. *Arch Pharm Res* **2004**, *27*, 217–224, doi:10.1007/BF02980109.
24. Hwang, J.; Kim, S.; Hwang, G.; Jeon, C.; Kang, K. Effect of Extract of Acanthopanax Senticosus Fruit on Breast Cancer Cells. *The Journal of Internal Korean Medicine* **2022**, *43*, 529–541, doi:https://doi.org/10.22246/jikm.2022.43.4.529.
25. Kou, X.; Li, Y.; Wang, L.; Song, X.; Li, D.; Wang, Z.; Zhao, Y.; Zhang, X.; Li, J.; Xing, Z. Apoptosis-Inducing Effects of Aqueous Extract of Eleutherococcus Senticosus on Non-Small Cell Lung Cancer Cell Proliferation. *Chinese Journal of Analytical Chemistry* **2025**, *53*, doi:10.1016/J.CJAC.2025.100510.
26. Zhang, J.Y. Experimental Intervention Effect of Acanthopanax Senticosus Polysaccharide on Lewis Lung Cancer in Mice. *Carcinogenesis, Teratogenesis and Mutagenesis* **2001**, *04*, 270.
27. Chen, X.J. The Anti-Tumor and Immune Effects of Acanthopanax Senticosus Polysaccharide. *Cancer* **1984**, *3*, 191–193.
28. Meng, Q.L. Basic Pharmacological Research on the Development of Changbai Mountain Acanthopanax Senticosus Polysaccharide. **2018**.
29. Meng, Q.; Pan, J.; Liu, Y.; Chen, L.; Ren, Y. Anti-Tumour Effects of Polysaccharide Extracted from Acanthopanax Senticosus and Cell-Mediated Immunity. *Exp Ther Med* **2018**, *15*, 1694–1701, doi:10.3892/etm.2017.5568.

30. Cao, G.W.; Du, P. Astragalus Polysaccharides, Acanthopanax Senticosus Polysaccharides and Lycium Barbarum Polysaccharides Regulate the Anti-Tumor Activity of LAK Cells in Vivo. *J. Second Mil. Med. Univ.* **1993**, *14*, 10–13.
31. Zhang, N.Z.; Zhang, W.Z.; Chen, X.J. Pathological Study of the Influence of Acanthopanax Senticosus Polysaccharide and GST on Experimental Liver Cancer. *Cancer Res.* **1997**, *02*, 78–80.
32. Bespalov, V.G.; Aleksandrov, V.A.; Semenov, A.L.; Kovan'ko, E.G.; Ivanov, S.D. Comparative Effects of Difluoromethylornithine and Siberian Ginseng Root Tincture on Radiation-Induced Carcinogenesis in Rats and Their Lifespan. *Advances in Gerontology* **2013**, *3*, 70–76, doi:10.1134/S2079057013010049.
33. Lee, C.H.; Huang, C.W.; Chang, P.C.; Shiau, J.P.; Lin, I.P.; Lin, M.Y.; Lai, C.C.; Chen, C.Y. Reactive Oxygen Species Mediate the Chemopreventive Effects of Syringin in Breast Cancer Cells. *Phytomedicine* **2019**, *61*, 152844, doi:10.1016/j.phymed.2019.152844.
34. Sun, D.; Chen, J.; Hu, H.; Lin, S.; Jin, L.; Luo, L.; Yan, X.; Zhang, C. Acanthopanax Senticosus Polysaccharide Suppressing Proliferation and Metastasis of the Human Non-Small Cell Lung Cancer NCI-H520 Cells Is Associated with Wnt/β-Catenin Signaling. *Neoplasma* **2019**, *66*, 555–563, doi:10.4149/neo.
35. Hibasami, H.; Fujikawa, T.; Takeda, H.; Nishibe, S.; Satoh, T.; Fujisawa, T.; Nakashima, K. Induction of Apoptosis by Acanthopanax Senticosus HARMS and Its Component, Sesamin in Human Stomach Cancer KATO III Cells. *Oncol Rep* **2000**, *7*, 1213–1216, doi:10.3892/or.7.6.1213.
36. Siao, A.C.; Hou, C.W.; Kao, Y.H.; Jeng, K.C. Effect of Sesamin on Apoptosis and Cell Cycle Arrest in Human Breast Cancer MCF-7 Cells. *Asian Pacific Journal of Cancer Prevention* **2015**, *16*, 3779–3783, doi:10.7314/APJCP.2015.16.9.3779.
37. Yamazaki, T.; Shimosaka, S.; Sasaki, H.; Matsumura, T.; Tukiyama, T.; Tokiwa, T. (+)-Syringaresinol-Di-O-β-d-Glucoside, a Phenolic Compound from Acanthopanax Senticosus Harms, Suppresses Proinflammatory Mediators in SW982 Human Synovial Sarcoma Cells by Inhibiting Activating Protein-1 and/or Nuclear Factor-KB Activities. *Toxicology in Vitro* **2007**, *21*, 1530–1537, doi:10.1016/j.tiv.2007.04.016.
38. Yamazaki, T.; Tokiwa, T. Isofraxidin, a Coumarin Component from Acanthopanax Senticosus, Inhibits Matrix Metalloproteinase-7 Expression and Cell Invasion of Human Hepatoma Cells. *Biol Pharm Bull* **2010**, *33*, 1716–1722, doi:10.1248/bpb.33.1716.
39. Zhao, J.X. Induces Apoptosis of Acanthopanax Senticosus Polysaccharides on H446 Cells. *Chinese Journal Cell Biology* **2018**, *02*, 239–242, doi:10.3969/j.issn.1674-7666.2008.02.021.
40. Zhao, J.X. ASPS Induces G_2/M Arrest of H446 Cells by Activation of ERK Signal Pathway. *Basic & Clinical Medicine* **2010**, *30*, 59–62.
41. Wang, F.F. *Study on the Structure and Chemical Modification of Acanthopanax Senticosus Polysaccharide*; Ocean University of China: Qingdao, 2006;
42. Wang, H.; Sun, B.; Zhang, Z.; Chen, J.; Hao, Q.; Sun, Y.; Yang, Y.; Wang, Z.; Pei, J. Effects of Acanthopanax Senticosus Polysaccharide on the Proliferation, Apoptosis and Cell Cycle in Human HepG2 Cells. *Pharmazie* **2016**, *71*, 201–204, doi:10.1691/ ph.2016.5807.
43. Jin, W. Experimental Study of Acanthopanax Senticosus Polysaccharide on the Proliferation and Apoptosis of Human Cervical Cancer HeLa Cells in Vitro. *Chin. Traditional Patent Med.* **2014**, *36*, 162–164.
44. Jin, W. Acanthopanax Senticosus Polysaccharide Down-Regulates Survivin Protein Expression and Induces HeLa Cell Apoptosis. *Chin. Traditional Patent Med.* **2016**, *38*, 902–904, doi:10.3969/j.issn.1001-1528.2016.04.040.
45. Tong, L. Experimental Study on the Anti-Tumor Effect and Mechanism of Acanthopanax Senticosus Polysaccharide. *Chin. Pharmacol. Bull.* **1994**, *10*, 105–109.

46. Chen, J.Y.; Yu, S.J.; Xiao, D. The Effection of Acanthopanax Senticosus Polysaccharide on Cancer Stem Cells. *Chin. J. Lab. Diagn.* **2019**, *23*, 1233–1238, doi:10.3969/j.issn.1007-4287.2019.07.04410.1177/ 1071100719879438.
47. Fu, J.; Yuan, J.; Tu, Y.; Fu, J.; Zhang, N.; Gao, B.; Fu, G.; Zhang, Y. A Polysaccharide from Acanthopanax Senticosus Improves the Antioxidant Status in Alloxan-Induced Diabetic Mice. *Carbohydr Polym* **2012**, *88*, 517–521, doi:10.1016/j.carbpol.2011.12.037.
48. Su, J.; Zhang, X.; Kan, Q.; Chu, X. Antioxidant Activity of Acanthopanax Senticosus Flavonoids in H2O2-Induced RAW 264.7 Cells and DSS-Induced Colitis in Mice. *Molecules* **2022**, *27*, doi:10.3390/molecules27092872.
49. Lee, S.; Son, D.; Ryu, J.; Yeon, S.L.; Sang, H.J.; Kang, J.; Sang, Y.L.; Kim, H.S.; Shin, K.H. Anti-Oxidant Activities of Acanthopanax Senticosus Stems and Their Lignan Components. *Arch Pharm Res* **2004**, *27*, 106–110, doi:10.1007/BF02980055.
50. Kim, Y.H.; Cho, M.L.; Kim, D.B.; Shin, G.H.; Lee, J.H.; Lee, J.S.; Park, S.O.; Lee, S.J.; Shin, H.M.; Lee, O.H. The Antioxidant Activity and Their Major Antioxidant Compounds from Acanthopanax Senticosus and A. Koreanum. *Molecules* **2015**, *20*, 13281–13295, doi:10.3390/molecules200713281.
51. Su, J.; Wang, Q.; Li, Z.; Feng, Y.; Li, Y.; Yang, S.; Feng, Y. Different Metabolites in the Roots, Seeds, and Leaves of Acanthopanax Senticosus and Their Role in Alleviating Oxidative Stress. *J Anal Methods Chem* **2021**, *2021*, doi:10.1155/2021/6628880.
52. Song, W.; Shi, J.; Baranenko, D.; Jing, J.; Lu, W. Radioprotective Effects of Active Compounds of Acanthopanax Senticosus from the Lesser Khingan Mountain Range in China Wei. *RSC Adv* **2016**, *6*, 65–72.
53. Meng, Q.F.; Yu, X.K.; Xu, M.Y.; Li, M.L.; Gao, Z.H.; Fan, H. Extraction of Acanthopanacis Senticosi Polysaccharides and Their Antioxidative Effect. *J. Jilin Univ. Inf. Sci. Ed.* **2005**, *43*, 683–686, doi:10.3321/ j.issn:1671-5489.2005.05.031.
54. Xie, Y.; Zhang, B.; Zhang, Y. Protective Effects of Acanthopanax Polysaccharides on Cerebral Ischemia–Reperfusion Injury and Its Mechanisms. *Int J Biol Macromol* **2015**, *72*, 946–950, doi:10.1016/J.IJBIOMAC.2014.09.055.
55. Liu, Y.; Huang, Q.C.; Cao, J.H. The Protective Effect of Acanthopanax Senticosus Polysaccharide on H2O2 Induced Apoptosis of Rat Hippocampal Neurons. *Chin. J. Clin. Neurosurg.* **2013**, *18*, 681–683.
56. Chen, R.; Liu, Z.; Zhao, J.; Chen, R.; Meng, F.; Zhang, M.; Ge, W. Antioxidant and Immunobiological Activity of Water-Soluble Polysaccharide Fractions Purified from Acanthopanax Senticosu. *Food Chem* **2011**, *127*, 434–440, doi:10.1016/j.foodchem.2010.12.143.
57. Diao, B.; Tang, Y.; Y.L. Zhu The Effect of Acanthopanax Senticosus Polysaccharide on the Expression of NF-Kb in Hippocampal Neurons Injured by H2O2. *Chin. J. Clin. Neurosurg.* **2010**, *15*, 350–352, doi:10.3969/j.issn.1009-153X.2010.06.011.
58. Diao, B. Effects of Acanthopanacis Senticosi Polysaccharides on Expression of INOS MRNA in Hippocampal Neurons Damaged by Oxidative Stress. *Military Medical Journal South China* **2008**, *19*, 15–17.
59. Jiang, Y.; Wang, M.H. Different Solvent Fractions of Acanthopanax Senticosus Harms Exert Antioxidant and Anti-Inflammatory Activities and Inhibit the Human Kv1.3 Channel. *J Med Food* **2015**, *18*, 468–475, doi:10.1089/jmf.2014.3182.
60. Zhang, N.; Zhao, L.-Y.; Mao, D.; Du, Z.-Y.; Zhang, X.-J.; Zhai, X.-N.; An, B.-S.; Liu, S.-M. Modulation Effect of Acanthopanax Senticosus Polysaccharides through Inflammatory Cytokines in Protecting Immunological Liver-Injured Mice [Article in Chinese]. *Zhongguo Zhong Yao Za Zhi* **2019**, *44*, 2947–2952, doi:10.19540/j.cnki.cjcmm.20190129.001.

61. Liu, L.; Mu, Q.; Li, W.; Xing, W.; Zhang, H.; Fan, T.; Yao, H.; He, L. Isofraxidin Protects Mice from LPS Challenge by Inhibiting Pro-Inflammatory Cytokines and Alleviating Histopathological Changes. *Immunobiology* **2015**, *220*, 406–413, doi:10.1016/j.imbio.2014.10.007.
62. He, C.; Chen, X.; Zhao, C.; Qie, Y.; Yan, Z.; Zhu, X. Eleutheroside E Ameliorates Arthritis Severity in Collagen-Induced Arthritis Mice Model by Suppressing Inflammatory Cytokine Release. *Inflammation* **2014**, *37*, 1533–1543, doi:10.1007/s10753-014-9880-7.
63. Xie, S.S. Immunoregulatory Effect of Polysaccharide of Acanthopanax Senticosus (PAS). I. Immunological Mechanism of PAS against Cancer [Article in Chinese]. *Zhonghua Zhong Liu Za Zhi* **1989**, *11*, 338–340.
64. Xie, S.S. Effect of Acanthopanax Senticosus Polysaccharide on Immune Function Reconstruction in Mice with Allogeneic Bone Marrow Transplantation. *J. Beijing Med. Univ* **1989**, *04*, 289–291.
65. Xu, S.K. Effect of Acanthopanax Senticosus Polysaccharide (ASPS) on Immune Function of Mice. *Chin. Patent Med.* **1990**, *03*, 25–26.
66. Luo, Q. Effects of Acanthopanax Senticosus Polysaccharide on Immune Function of Normal Mice. *Heilongjiang Anim. Sci. Vet. Med.* **2013**, *13*, 120–122.
67. Luo, Q. Effects of Acanthopanax Senticosus Polysaccharide on Lymphocyte Transformation Rate and Peripheral Lymphocyte Subsets in Normal Mice. *J. Henan Agric. Sci.* **2013**, *42*, 137–140.
68. Zhai, X.N. Effect of Acanthopanax Senticosus Polysaccharide on Immune Function of Mice. *Traditional Chin. Med. Inf.* **2020**, *37*, 42–45, doi:doi:10.19656/ j.cnki.1002-2406.200158.
69. Sun, S.K.; Song, T.; Y. Sun Immunomodulatory Effects of Acanthopanax Senticosus Acidic Polysaccharides in Cyclophosphamide- Induced Immunocompromised Mice. *Immunological J.* **2018**, *34*, 863–868, doi:10.13431/j.cnki.immunol.j.20180133.
70. Yang, S.; Shan, C.; Ma, X.; Qin, Y.; Ju, A.; Duan, A.; Luan, W.; Zhang, Y. Immunomodulatory Effect of Acanthopanax Senticosus Polysaccharide on Immunosuppressed Chickens. *Poult Sci* **2021**, *100*, 623–630, doi:10.1016/j.psj.2020.11.059.
71. Lin, Q.Y.; Jin, L.J.; Cao, Z.H.; Li, H.Q.; Xu, Y.P. Protective Effect of Acanthopanax Senticosus Extract against Endotoxic Shock in Mice. *J Ethnopharmacol* **2008**, *118*, 495–502, doi:10.1016/j.jep.2008.05.018.
72. Lau, K.M.; Yue, G.G.L.; Chan, Y.Y.; Kwok, H.F.; Gao, S.; Wong, C.W.; Lau, C.B.S. A Review on the Immunomodulatory Activity of Acanthopanax Senticosus and Its Active Components. *Chinese Medicine (United Kingdom)* **2019**, *14*, 1–6, doi:10.1186/s13020-019-0250-0.
73. Han, S.B.; Yoon, Y.D.; Ahn, H.J.; Lee, H.S.; Lee, C.W.; Yoon, W.K.; Park, S.K.; Kim, H.M. Toll-like Receptor-Mediated Activation of B Cells and Macrophages by Polysaccharide Isolated from Cell Culture of Acanthopanax Senticosus. *Int Immunopharmacol* **2003**, *3*, 1301–1312, doi:10.1016/S1567-5769(03)00118-8.
74. Panossian, A.; Davtyan, T.; Gukassyan, N.; Gukasova, G.; Mamikonyan, G.; Gabrielian, E.; Wikman, G. Effect of Andrographolide and Kan Jang - Fixed Combination of Extract SHA-10 and Extract SHE-3 - On Proliferation of Human Lymphocytes, Production of Cytokines and Immune Activation Markers in the Whole Blood Cells Culture. *Phytomedicine* **2002**, *9*, 598–605, doi:10.1078/094471102321616409.
75. Steinmann, G.G.; Esperester, A.; Joller, P. Immunopharmacological in Vitro Effects of Eleutherococcus Senticosus Extracts. *Arzneimittel-Forschung/Drug Research* **2001**, *51*, 76–83, doi:10.1055/s-0031-1300006.
76. Han, J.; Bian, L.; Liu, X.; Zhang, F.; Zhang, Y.; Yu, N. Effects of Acanthopanax Senticosus Polysaccharide Supplementation on Growth Performance, Immunity, Blood Parameters and Expression of pro-Inflammatory Cytokines Genes in Challenged Weaned Piglets. *Asian-Australas J Anim Sci* **2014**, *27*, 1035–1043, doi:10.5713/ajas.2013.13659.

77. Wang, Y.; Wang, L.; Zhang, H.; Ren, P.; Cheng, X.; Hong, F.; Liu, J.; Zhang, R.; Zhao, J.; Gou, D. Immunostimulatory Effects Mechanism of Polysaccharide Extracted from Acanthopanax Senticosus on RAW 264.7 Cells through Activating the TLR/MAPK/NF-KB Signaling Pathway. *Sci Rep* **2025**, *15*, 13440, doi:10.1038/s41598-025-97423-3.
78. Luo, Q. Study on Cell K562 Apoptosis Induced by Acanthopanax Senticosus in Vitro. *Journal Hebei North University (Medical Edition)* **2008**, *05*, 17–19.
79. Wang, X.; Zhou, G.; Liu, C.; Wei, R.; Zhu, S.; Xu, Y.; Wu, M.; Miao, Q. Acanthopanax versus 3-Methyladenine Ameliorates Sodium Taurocholate-Induced Severe Acute Pancreatitis by Inhibiting the Autophagic Pathway in Rats. *Mediators Inflamm* **2016**, *2016*, doi:10.1155/2016/8369704.
80. Wang, X.; Zhang, X.; Su, J.; Chu, X. Acanthopanax Senticosus Total Flavonoids Alleviate Lipopolysaccharide-Induced Intestinal Inflammation and Modulate the Gut Microbiota in Mice. *Biosci Rep* **2022**, *42*, doi:https://doi.org/10.1042/BSR20212670.
81. Fei, X.J.; Zhu, L.L.; Xia, L.M.; Peng, W.B.; Wang, Q. Acanthopanax Senticosus Attenuates Inflammation in Lipopolysaccharide-Induced Acute Lung Injury by Inhibiting the NF-KB Pathway. *Genetics and Molecular Research* **2014**, *13*, 10537–10544, doi:10.4238/2014.December.12.16.
82. Zhang, N. Modulation of Acanthopanax Senticosus Polysaccharides on Expression of IL-2,IL-4, INF-γ Cytokines, and MRNA in BALB/c Immunological Liver-Injured Mice. *Drug Eval. Res.* **2018**, *41*, 557–561, doi:10.7501/j.issn.1674-6376.2018.04.010.
83. Yang, X.D. *Protective Effects of Acanthopanax Senticosus Polysaccharide on Immune Liver Injury in Mice*; Heilongjiang University of Traditional Chinese Medicine: Harbin, 2016;
84. Zhang, H.; Wang, S.; Jin, L.H. Acanthopanax Senticosus Polysaccharide Regulates the Intestinal Homeostasis Disruption Induced by Toxic Chemicals in Drosophila. *Phytotherapy Research* **2020**, *34*, 193–200, doi:10.1002/ptr.6522.
85. Han, J.; Li, J.H.; Bai, G.; Shen, G.S.; Chen, J.; Liu, J.N.; Shuo, W.; Liu, X.J. Acanthopanax Senticosus Polysaccharides-Induced Intestinal Tight Junction Injury Alleviation via Inhibition of NF-KB/MLCK Pathway in a Mouse Endotoxemia Model. *World J Gastroenterol* **2017**, *23*, 2175–2184, doi:10.3748/wjg.v23.i12.2175.
86. Lu, F. Investigation of Protective Effect of Acanthopanax Senticosus Polysaccharides on Immunological Liver Injury in Mice by Metabolomics Methods. *New Chin. Med. Clin. Pharmacol.* **2016**, *27*, 823–829, doi:10.19378/j.issn.1003-9783.2016.06.016.
87. Guan, S.; Ma, J.; Chu, X.; Gao, Y.; Zhang, Y.; Zhang, X.; Zhang, F.; Liu, Z.; Zhang, J.; Chu, L. Effects of Total Flavones from Acanthopanax Senticosus on L-Type Calcium Channels, Calcium Transient and Contractility in Rat Ventricular Myocytes. *Phytotherapy Research* **2015**, *29*, 533–539, doi:10.1002/ptr.5278.
88. Wang, R.; Shi, L.; Liu, S.; Liu, Z.; Song, F.; Sun, Z.; Liu, Z. Mass Spectrometry-Based Urinary Metabolomics for the Investigation on the Mechanism of Action of Eleutherococcus Senticosus (Rupr. & Maxim.)Maxim. Leaves against Ischemic Stroke in Rats. *J Ethnopharmacol* **2019**, *241*, 111969, doi:10.1016/j.jep.2019.111969.
89. Sui, D.Y.; Qu, S.C.; Yu, X.F.; Chen, Y.P.; X.Y. Ma Protective Effect of ASS on Myocardial Ischemia-Reperfusion Injury in Rats. *Zhongguo Zhong Yao Za Zhi* **2004**, *29*, 71–74.
90. Liang, Q.; Yu, X.; Qu, S.; Xu, H.; Sui, D. Acanthopanax Senticosides B Ameliorates Oxidative Damage Induced by Hydrogen Peroxide in Cultured Neonatal Rat Cardiomyocytes. *Eur J Pharmacol* **2010**, *627*, 209–215, doi:10.1016/j.ejphar.2009.10.055.
91. Zhou, Y.; Cheng, C.; Baranenko, D.; Wang, J.; Li, Y.; Lu, W. Effects of Acanthopanax Senticosus on Brain Injury Induced by Simulated Spatial Radiation in Mouse Model Based on Pharmacokinetics and Comparative Proteomics. *Int J Mol Sci* **2018**, *19*, doi:10.3390/ijms19010159.

92. Fujikawa, T.; Yamaguchi, A.; Morita, I.; Takeda, H.; Nishibe, S. Protective Effects of Acanthopanax Senticosus Harms from Hokkaido and Its Components on Gastric Ulcer in Restrained Cold Water Stressed Rats. *Biological Pharmaceutical Bulletin* **1996**, *19*, 1227–1230.
